# Association between trajectory of triglyceride-glucose index and all-cause mortality in critically ill patients with atrial fibrillation: a retrospective cohort study

**DOI:** 10.1186/s12933-025-02838-x

**Published:** 2025-07-10

**Authors:** Shangsong Shi, Feng Xue, Tingbo Jiang, Lin Ling

**Affiliations:** Department of Cardiology, The First Affiliated Hospital of Soochow University, Suzhou City, 215006 Jiangsu Province China

**Keywords:** Atrial fibrillation, Triglyceride-glucose index trajectory, Intensive care unit, All-cause mortality, MIMIC database

## Abstract

**Introduction:**

Previous evidence showed that triglyceride-glucose (TyG) index is strongly associated with poor prognosis in atrial fibrillation (AF) in the general population. In critically ill patients, physiological stress may cause rapid fluctuation in the TyG index, making single measurements insufficient for prognosis assessment. Furthermore, the impact of TyG index trajectories on outcomes in critically ill patients with atrial fibrillation has not yet been well elucidated. Therefore, our study aimed to assess the association between TyG index trajectories in patients with AF in intensive care unit (ICU) and all-cause mortality at 30-day, 90-day, 180-day and 365-day follow-up.

**Methods:**

We used data from Medical Information Mart for Intensive Care (MIMIC)-IV database. Patients diagnosed with AF in ICU were enrolled. We applied group-based trajectory modeling to identify distinct TyG index trajectories, selecting the optimal model based on the Bayesian information criterion (BIC), Akaike information criterion (AIC), average posterior probability (AvePP), and clinical interpretability. Kaplan-Meier survival curve was used to compare the mortality in AF patients with different TyG index trajectories. Hazard ratios (HRs) were calculated to elucidate the association between trajectories and prognosis in Cox proportional hazard models. Restricted cubic splines (RCS) were used to assess the relationship between TyG index and outcomes.

**Results:**

A total of 1,108 AF patients were enrolled. Four TyG index trajectories were identified including: (1) traj1 group (TyG index stable at low level), (2) traj2 group (TyG index slowly ascending at moderate level), (3) traj3 group (TyG index ascending then descending at moderate high level) and (4) traj4 group (TyG index fluctuate at high level). The Traj4 group demonstrated significantly higher mortality rates at all time points (30-day, 90-day, 180-day and 365-day) compared to other trajectory groups. In addition, Cox proportional hazard models indicated that patients in traj4 group had higher risk of mortality compared to those in traj1 group at 30-day (HR = 1.71, 95% confidence interval [CI], 1.14–2.56), 90-day (HR = 1.67, 95% CI, 1.17–2.39), 180-day (HR = 1.44, 95% CI, 1.03–2.06) and 365-day (HR = 1.44, 95% CI, 1.04–1.98). Meanwhile, the RCS indicated a linear association between TyG index and all-cause mortality.

**Conclusion:**

In critically ill patients with AF, TyG index trajectories were significantly associated with 30-day, 90-day, 180-day and 365-day all-cause mortality. This suggested that TyG index trajectories could serve as a robust indicator for risk stratification and prognosis assessment in ICU patients with AF.

**Graphical Abstract:**

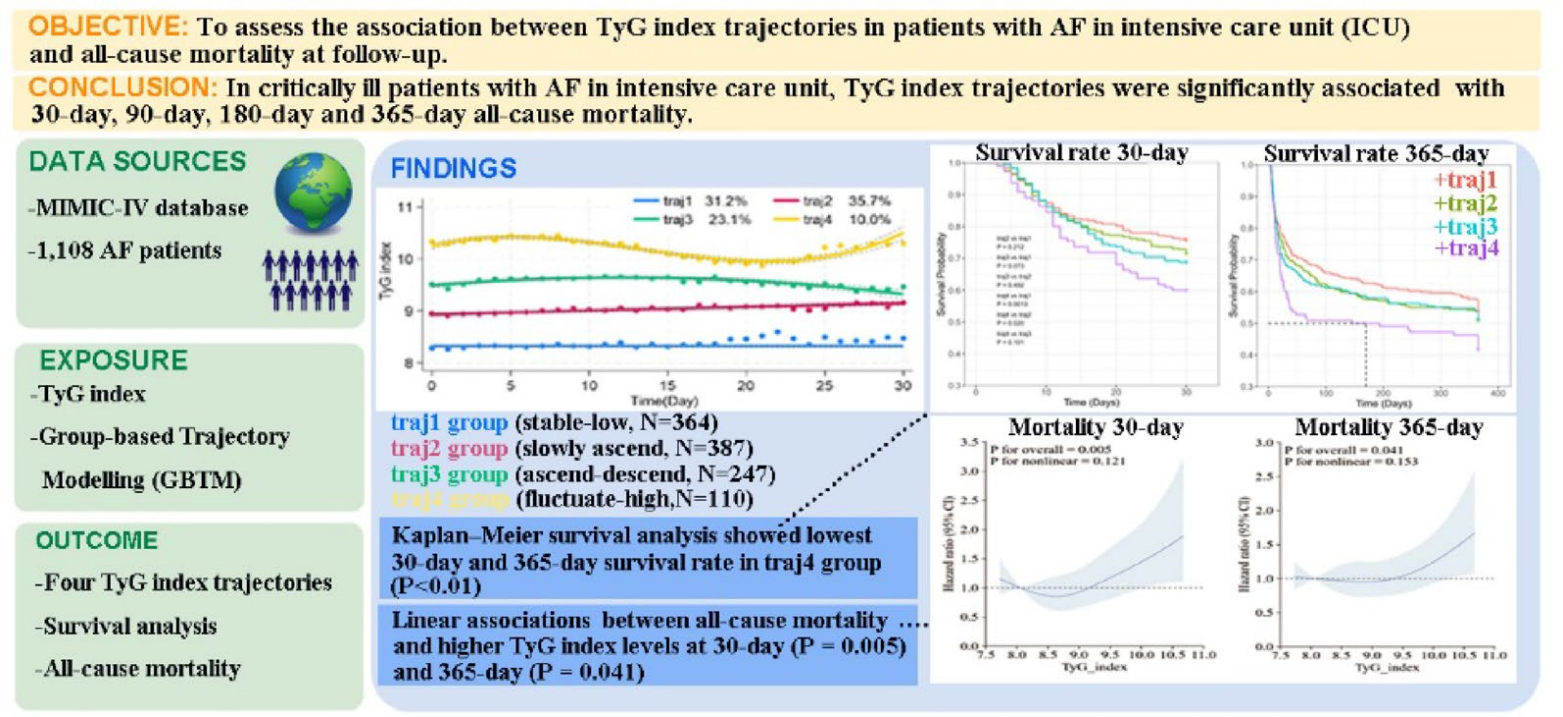

**Supplementary Information:**

The online version contains supplementary material available at 10.1186/s12933-025-02838-x.

## Introduction

Atrial fibrillation (AF) is the most common cardiac arrhythmia worldwide, and brings increasing healthy and economic global burden. It is associated with increased risks of stroke, cognitive impairment, myocardial infarction (MI), heart failure (HF) and sudden cardiac death [1, 2]. Meanwhile, AF is also the most common arrhythmia in the intensive care unit (ICU), and according to an international cohort study, the incidence of AF in ICU was 15.6%. Compared to those without AF, patients with AF tend to be associated with poor prognosis [3, 4]. However, studies which demonstrate the prognosis of critically ill patients with AF in the ICU are still insufficient. Therefore, finding an effective and non-invasive method to identify the mortality in critically ill patients with AF is important.

Insulin resistance (IR) is a pathological condition characterized by diminished responses to insulin and hyperinsulinemia. IR is the typical feature of metabolic syndrome and type 2 diabetes, and has been discovered to link to cardiovascular stiffening, which leads to obesity, cardiovascular disease, kidney disease and so on [5–7]. So far, numerous studies have reported the relationship between IR and AF. The hyperinsulinemic-euglycemic clamp (HEC) test, the gold standard for insulin resistance (IR) measurement, along with its alternative method—the homeostatic model assessment of insulin resistance (HOMA-IR), have limited clinical applications due to their complexity, time-consuming procedures and high costs. Currently, the triglyceride-glucose (TyG) index has been widely validated as a novel and simple surrogate marker for IR assessment. Moreover, studies have demonstrated its diagnostic and prognostic value in patients with AF [8, 9]. Previous studies have validated that higher glucose levels in the ICU are associated with increased mortality, and early glucose control or intensive insulin therapy may improve patient prognosis. These findings suggest that insulin resistance is closely linked to mortality in ICU settings [10–12]. The TyG index, as an indicator of insulin resistance, has been shown to correlate strongly with disease prognosis in critically ill patients [13–15].

Previous studies have validated TyG index as a strong predictor of prognosis in critically ill patients with AF. However, these studies only focused on a single-measurement of the TyG index, neglecting the influence of dynamic TyG index trajectories, which represent changes in IR or hyperglycemia status. This is particularly important for ICU patients, as they are always in a stress response state and the insulin resistance can change over time [16–18]. In critically ill patients, physiological stress may cause rapid fluctuation in the TyG index, rendering single measurements insufficient for prognosis assessment. The dynamic changes in insulin resistance are typically reflected by changes in glucose levels. Currently, we can utilize the TyG index trajectory to precisely evaluate these changes. Previous studies have shown that dynamic changes in glucose or insulin resistance are significantly associated with mortality in ICU patients [19–22]. However, researches investigating the relationship between the dynamic changes in the TyG index and all-cause mortality among AF patients in the ICU remain limited.

In summary, our study aims to investigate the association between dynamic TyG index trajectories and all-cause mortality in critically ill patients with AF. Our goal is to assess the clinical value of TyG index trajectories as a reliable and simplified prognostic marker in this population, thereby providing robust evidence to guide clinical decision-making in intensive care settings.

## Methods

### Source of data

This study is a retrospective analysis using data from the publicly available Medical Information Mart for Intensive Care IV (MIMIC-IV, version 3.1) database. The MIMIC-IV database, with several improvements over MIMIC-III, contains data of over 65,000 patients admitted to the ICU and over 200,000 patients admitted to the emergency department, recorded from 2008 to 2022 at the Beth Israel Deaconess Medical Center (BIDMC, Boston, MA, United States). The database records detailed information of patients’ demographics, laboratory tests, medications, vital signs, surgical operations, disease diagnosis, medication management, and follow-up survival status. In order to obtain data access, we have completed the training course of National Institutes of Health (NIH) for protecting human study participants and passed the tests of the Collaborative Institutional Training Initiative. The author Shangsong Shi has completed the Collaborative Institutional Training Program exam (certification number: 65,769,961) and the research resource has received institutional review board (IRB) approval at the BIDMC. Informed consent is not necessary for the secondary utilization of this de-identified database [16, 23].

### Study design and population

**Fig. 1:**
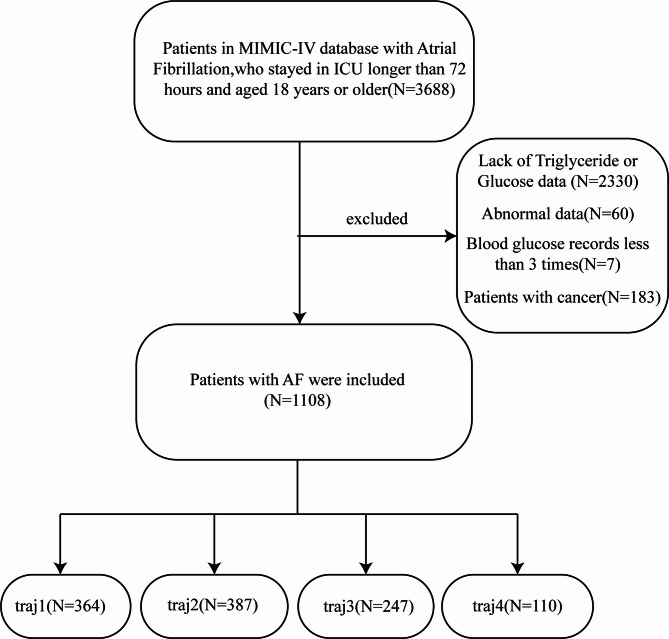
Flowchart of the selection of patients

Our study included 3,688 patients with AF who were admitted to the ICU for the first time and aged 18 years or older. According to International Classification of Diseases (ICD)-9 or ICD-10 codes (42731, I48, I480, I481, I4811, I4819, I482, I4820, I4821, I489, I4891), we selected all AF patients in MIMIC-IV. To establish reliable dynamic trajectories, we required patients to have an intensive care unit (ICU) length of stay (LOS) exceeding 72 h. This criterion was necessary because (1) blood glucose and triglyceride measurements are typically not performed multiple times per day and (2) constructing valid TyG index trajectories requires at least three measurement time points. We excluded patients based on the following criteria: (1) ICU length of stay less than 72 h; (2) non-adult patients with age below 18 years; (3) for patients with multiple ICU admissions, only data from the first admission were extracted; (4) blood glucose levels were measured fewer than 3 times during the ICU stay; (5) insufficient data (such as blood glucose, serum triglycerides and other major data); (6) patients with cancer. Totally, 1,108 patients met the inclusion criteria (Fig. 1).

### Data extraction

We used Navicat Premium (Version 16.3.2) with Structured Query Language (SQL) to extract data. All laboratory parameters (except blood glucose and triglycerides) and vital signs were measured during the first 24 h following ICU admission. For indicators with multiple recordings during this period, we calculated and used the mean values. Regarding glucose and triglyceride measurements, we established a 24-h time window for data pairing starting from ICU admission. To minimize potential ICU-related fluctuations, we selected the lowest daily recorded value as an independent data point. Medication history was extracted based on documented administration records during the ICU stay.

Numerous data for each patient at admission were extracted, including demographic information (age, race, gender, weight); vital sign (heart rate [HR], systolic blood pressure [SBP], diastolic blood pressure [DBP], mean blood pressure [MBP], respiratory rate [RR]); Glasgow coma scale [GCS], simplified acute physiology score [SAPS II], sequential organ failure assessment [SOFA]; Charlson comorbidity index; Oxford Acute Severity of Illness Score (OASIS); past history (acute myocardial infarction [AMI], hypertension, diabetes, chronic obstructive pulmonary disease [COPD], congestive heart failure [CHF], peripheral vascular disease [PVD], cerebrovascular disease [CVD], rheumatic disease, liver disease, sepsis); medications (aspirin, clopidogrel, beta-blockers, angiotensin converting enzyme inhibitors [ACEI], calcium channel blocker [CCB], digitalis, amiodarone, insulin, statin, heparin, warfarin); mean laboratory data (SpO2, anion gap, bicarbonate, glucose, blood urea nitrogen [BUN], creatinine, calcium, chloride, sodium, potassium, prothrombin time [PT], partial prothrombin time [PTT], international normalized ratio [INR], hematocrit, hemoglobin, platelets, white blood cell [WBC], mean corpuscular hemoglobin [MCH], mean corpuscular hemoglobin concentration [MCHC], mean corpuscular volume [MCV], red blood cell [RBC], red cell distribution width [RDW], triglyceride [TG]); length of stay (LOS) in ICU; outcomes (30-day mortality, 90-day mortality, 180-day mortality and 365-day mortality). Variables with more than 10% missing values were excluded. Variables with missing values less than 10% were imputed using multiple interpolation. The main predictor was the in-ICU TyG index trajectory, which was assessed for each patient based on the TyG index values during their ICU stay.

### Outcome

The primary outcomes of our study were 30-day and 365-day all-cause mortality, and the secondary outcomes included 90-day and 180-day all-cause mortality.

### Calculation of TyG index

The TyG index was calculated by the formula ln(fasting triglycerides [mg/dL] × fasting glucose [mg/dL]/2).

### Statistical analysis

For continuous variables, we executed the Shapiro–Wilk test to determine whether the data were normally distributed, those normally distributed variables were presented as mean ± standard deviation (SD), otherwise, data were presented as median with interquartile range (IQR). Categorical variables were presented as absolute counts (percentages). If the data exhibited a normal distribution across groups and the homogeneity of variance test revealed no statistically significant differences, we used the one-way ANOVAs for continuous variables, otherwise, we employed the Kruskal–Wallis test for continuous variables. Chi-square test were used for categorical variables.

TyG index trajectories during ICU stay were generated using Group-based Trajectory Modelling (GBTM), implemented in Stata (version 18.0) with the “Traj” package, to categorize longitudinal measurements into interrelated subgroups. GBTM predicted trajectories for each group, estimated each individual’s probability of group membership, and assigned individuals to groups based on their highest probabilities, which were modeled by a finite set of different polynomial time functions. Then, we identified the optimal number of trajectory classes using improvement in the absolute value of Bayesian Information Criterion (BIC) values and Akaike Information Criterion (AIC), assignment probabilities (> 70%), minimal class sizes (> 5% of sample size) and clinical experience (Table 1) [24].

**Table 1:** The Group-based Trajectory Modelling (GBTM) parameters for triglyceride-glucose index trajectory grouping

Number of classes	Log likelihood	AIC	BIC	AvePP	Class 1 (%)	Class 2 (%)	Class 3 (%)	Class 4 (%)	Class 5 (%)
1	-16,204.97	-16,208.97	-16,219.30	1.00	100				
2	-11,697.40	-11,705.40	-11,726.05	0.98	63.67	36.33			
3	-9610.71	-9622.71	-9653.69	0.97	40.90	41.36	17.75		
4	-8709.56	-8723.56	-8759.70	0.96	31.19	35.71	23.07	10.03	
5	-8269.90	-8289.90	-8341.53	0.94	20.56	29.11	25.23	16.11	8.98

AIC, Akaike Information Criterion; BIC, Bayesian Information Criteria; AvePP, Average Posterior Probabilities

**Fig. 2:**
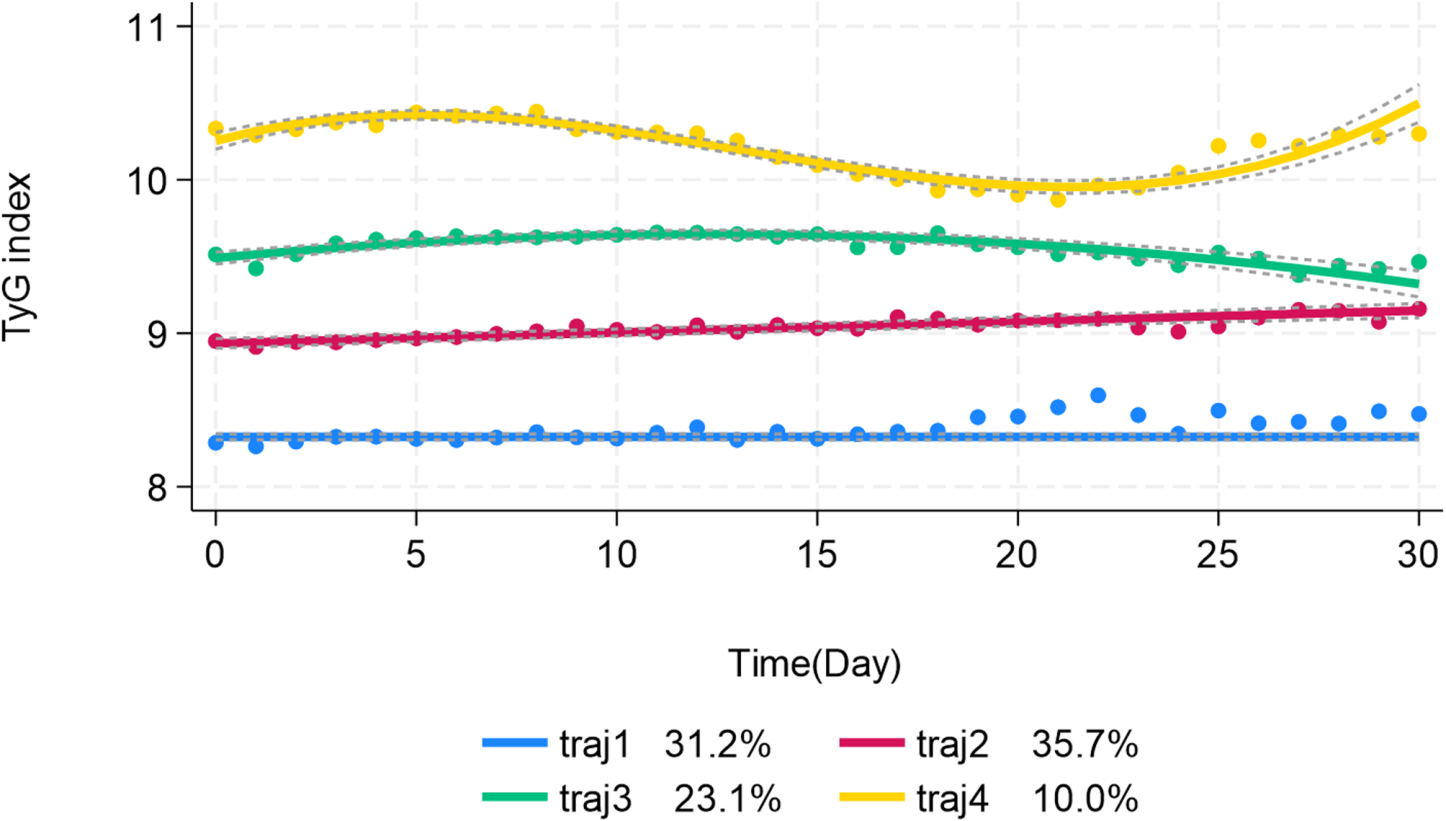
Identification of triglyceride-glucose index trajectories. traj1(31.2%): stable-low group; traj2(35.7%): slowly ascend group; traj3(23.1%): ascend-descend group; traj4(10.0%): fluctuate-high group

Based on our selection criteria, we chose the 4-class model as the final model because it demonstrated a smaller improvement in the absolute values of BIC and AIC, had assignment probabilities exceeding 70%, and offered stronger clinical interpretability compared to the 5-class model. Then, TyG index were categorized into four main trajectories: (1) traj1 group, with TyG index stable in relatively low level, (2) traj2 group, with TyG index slowly ascending in relatively moderate level, (3) traj3 group, in which TyG index ascending then descending in relatively moderate high level and (4) traj4 group, in which TyG index fluctuate in high level. All trajectories were shown in Fig. 2 [24, 25].

Survival analysis was conducted using the Kaplan–Meier curve with the log-rank test based on TyG index trajectories at 30-day mortality, 90-day mortality, 180-day mortality and 365-day mortality. Furthermore, we conducted Univariable Cox regression analysis to select confounding variables related to the outcome. Cox proportional hazard models were performed to calculate the hazard ratios (HRs) and 95% confidence intervals (CIs) and the association between TyG index trajectories and 30-day, 90-day, 180-day and 365-day mortality of AF patients were assessed. Confounding variables were selected by the following criteria: (1) statistical significance (*p* < 0.05 in univariate analysis) and (2) clinical relevance (variables that altered the hazard ratio by > 10% or had established clinical importance). Ultimately, we constructed three adjusted models to account for potential confounding covariates: Crude model was unadjusted by any confounders. Model I was adjusted by age, gender, race, heart rate, weight. Model II was adjusted by amiodarone, congestive heart failure, liver disease, diabetes, statin, sepsis, hypertension, beta-blocker, WBC, RBC, BUN, RDW, creatinine and all variables in Model I. Restricted cubic splines (RCS) were utilized to explore the potential linear correlation between TyG index and major outcome events after adjusted these same covariates in Model II and we specified four knots for the RCS analysis. We also conducted subgroup analysis of age, gender, race, sepsis, liver disease, diabetes, usage of statin and insulin to explore the relationship between TyG index trajectories and 30-day, 90-day, 180-day and 365-day mortality. Bonferroni correction was used to correct alpha error inflation and interaction testing was conducted in our subgroup analysis. Statistical analyses in this study were conducted using R Studio (version R 4.2.3), A two-sided P-value of < 0.05 was considered statistically significant.

## Results

### Baseline characteristics

In this study, a total of 1,108 AF patients were enrolled from the MIMIC-IV database. The flowchart shown in Fig. 1 presented how patients were selected for this study. GBTM distributed different trajectory model probabilities to every patients. Based on the highest trajectory probability, patients were divided into four distinct trajectory groups: traj1 group (stable-low, N = 364), traj2 group (slowly ascend, N = 387), traj3 group (ascend-descend, N = 247) and traj4 group (fluctuate-high, N = 110). Baseline characteristics of the total population and groups stratified by TyG index trajectory groups were presented in Table 2. The traj1 group patients with the lowest average TyG index level were older, lighter in weight and contained more percentages of female. They showed a higher Charlson Comorbidity Index, higher prevalence of cerebrovascular disease, peripheral vascular disease, hypertension and congestive heart failure. They exhibited lower prevalence of diabetes and sepsis, lower usage rate of amiodarone, heparin, beta blocker, CCB and insulin. They also had higher blood level of calcium, bicarbonate, sodium and blood pressure, lower level of WBC counts, anion gap, BUN, creatinine, potassium, lower score of SOFA, APSIII, GCS, OASIS and SAPS II compared with patients in higher TyG index trajectory groups.

**Table 2:** Baseline characteristics of patients grouped according to triglyceride glucose index trajectory

Variables	Total(N = 1,108)	Traj1(N = 364)	Traj2(N = 387)	Traj3(N = 247)	Traj4(N = 110)	P-value
Age,Years	73.7 [64.7, 82.0]	77.5 [68.6, 85.9]	74.3 [66.1, 82.0]	69.6 [61.1, 77.3]	67.4 [59.1, 74.2]	** < 0.001**
Race,white(%)	566 (51.1)	192 (52.2)	207 (53.5)	110 (44.5)	57 (51.8)	0.135
Female,N(%)	430 (38.8)	159 (43.7)	150 (38.8)	89 (36.0)	32 (29.1)	**0.032**
Weight,Kg	83.05 [69.0, 100.8]	75.05 [63.5, 90.2]	83.00 [69.3, 102.0]	90.00 [73.0, 106.6]	94.00 [82.6, 119.8]	** < 0.001**
HR,Beats/min	85 [74, 98]	81 [72, 95]	85 [75, 98]	86 [75, 98]	87 [76, 100]	**0.044**
SBP,mmHg	116 [107, 130]	118 [105, 133]	116 [106, 130]	115 [107, 129]	114 [108, 124]	0.525
DBP,mmHg	64 [57, 72]	65 [58, 75]	64 [57, 73]	64 [57, 71]	62 [56, 69]	0.074
MBP,mmHg	80 [73, 88]	80 [74, 91]	80 [73, 89]	80 [73, 86]	77 [73, 83]	0.118
RR,Times/min	20 [18, 23]	19 [17, 22]	20 [18, 23]	20 [18, 24]	23 [20,26]	** < 0.001**
Spo2,%	97 [95, 98]	97 [95, 98]	97 [95, 98]	97 [95, 98]	96 [94, 97]	**0.001**
SOFA score	6.0 [3.0, 9.0]	4.0 [3.0, 7.0]	5.0 [3.0, 8.5]	7.0 [4.0, 10.0]	8.0 [5.3, 11.0]	** < 0.001**
APSIII score	49.0 [37.0, 65.0]	43.0 [34.0, 57.0]	49.0 [36.0, 64.0]	50.0 [39.0, 67.0]	62.0 [50.0, 83.8]	** < 0.001**
GCS score	15.0 [14.0, 15.0]	15.0 [14.0, 15.0]	15.0 [15.0, 15.0]	15.0 [15.0, 15.0]	15.0 [15.0, 15.0]	** < 0.001**
OASIS score	35.5 [30.0, 42.0]	34.0 [29.0, 40.0]	35.0 [30.0, 42.0]	36.0 [31.5, 42.0]	39.5 [34.3, 45.0]	** < 0.001**
SAPS II score	41.0 [33.0, 51.0]	38.0 [32.0, 48.0]	40.0 [33.0, 51.0]	43.0 [33.5, 53.0]	47.0 [38.0, 58.8	** < 0.001**
Charlson Comorbidity Index	6.0 [4.0, 8.0]	7.0 [5.0, 8.0]	6.0 [5.0, 8.0]	6.0 [4.0, 7.5]	5.0 [3.0, 7.8]	** < 0.001**
Myocardial Infarct,N(%)	282 (25.5)	86 (23.6)	108 (27.9)	61 (24.7)	27 (24.6)	0.574
Congestive Heart Failure,N(%)	540 (48.7)	184 (50.6)	201 (51.9)	113 (45.8)	42 (38.2)	**0.049**
Peripheral Vascular Disease,N(%)	160 (14.4)	57 (15.7)	49 (12.7)	37 (15.0)	17 (15.5)	0.664
Cerebrovascular Disease,N(%)	502 (45.3)	199 (54.7)	184 (47.6)	93 (37.7)	26 (23.6)	** < 0.001**
Hypertension,N(%)	383 (34.6)	134 (36.8)	135 (34.9)	80 (32.4)	34 (30.9)	0.573
Chronic Pulmonary Disease,N(%)	238 (21.5)	63 (17.3)	88 (22.7)	56 (22.7)	31 (28.2)	0.064
Rheumatic Disease,N (%)	40 (3.6)	10 (2.8)	14 (3.6)	12 (4.9)	4 (3.6)	0.597
Liver Disease,N(%)	129 (11.6)	43 (11.8)	52 (13.4)	23 (9.3)	11 (10.0)	0.421
Diabetes,N(%)	185 (16.7)	38 (10.4)	73 (18.7)	51 (20.7)	23 (20.9)	**0.001**
Renal Disease,N(%)	324 (29.2)	100 (27.5)	119 (30.8)	72 (29.2)	33 (30.0)	0.799
Sepsis,N(%)	785 (70.6)	207 (56.9)	271 (70.0)	204 (82.6)	103 (93.6)	** < 0.001**
ACEI,N(%)	43 (3.9)	17 (4.7)	20 (5.2)	4 (1.6)	2 (1.8)	0.073
Amiodarone,N(%)	219 (19.8)	45 (12.4)	78 (20.2)	54 (21.9)	42 (38.2)	** < 0.001**
Aspirin,N(%)	565 (51.0)	183 (50.3)	209 (54.0)	127 (51.4)	46 (41.8)	0.158
Clopidogrel,N(%)	97 (8.8)	31 (8.5)	37 (9.6)	24 (9.7)	5 (4.6)	0.381
Heparin,N(%)	444 (40.1)	120 (33.0)	162 (41.9)	106 (42.9)	56 (50.9)	**0.002**
Warfarin,N(%)	12 (1.1)	4 (1.1)	5 (1.3)	3 (1.2)	0 (0.0)	0.705
Statin,N(%)	624 (56.3)	203 (55.8)	226 (58.4)	138 (55.9)	57 (51.8)	0.648
Beta blocker,N(%)	374 (33.8)	100 (27.5)	147 (38.0)	82 (33.2)	45 (40.9)	**0.007**
Digoxin,N(%)	47 (4.2)	8 (2.2)	21 (5.4)	12 (4.9)	6 (5.5)	0.127
CCB,N(%)	236 (21.3)	60 (16.5)	99 (25.6)	50 (20.2)	27 (24.6)	**0.018**
Insulin,N(%)	485 (43.8)	106 (29.1)	174 (45.0)	127 (51.4)	78 (70.9)	** < 0.001**
RBC,m/μL	3.7 [3.2, 4.3]	3.7 [3.2, 4.3]	3.7 [3.1, 4.3]	3.7 [3.1, 4.3]	3.8 [3.3, 4.3]	0.83
WBC,K/μL	11.6 [8.7, 15.7]	10.1 [8.1, 13.6]	12.2 [8.9, 15.9]	12.6 [9.2, 17.0]	13.8 [9.5, 19.7]	** < 0.001**
Hematocrit,%	34.4 [29.3, 39.1]	34.3 [29.7, 38.7]	34.5 [28.8, 39.5]	34.1 [29.1, 39.2]	34.8 [30.4, 40.0]	0.973
Hemoglobin,g/dL	11.0 [9.4, 12.8]	11 [9.4, 12.6]	11.1 [9.2, 12.9]	11.0 [9.4, 12.8]	11.12 [9.8, 12.5]	0.984
Platelet,K/uL	181.0 [135.0, 242.3]	178.2 [134.9, 240.4]	179.2 [132.8, 240.0]	191.0 [143.0, 243.5]	188.9 [132.3, 258.8]	0.503
MCH,pg	30.0 [28.6, 31.6]	30.1 [28.6, 31.8]	30.1 [28.9, 31.6]	29.9 [28.6, 31.3]	29.6 [27.8, 31.4]	0.215
MCHC,%	32.4 [31.6, 33.3]	32.5 [31.6, 33.2]	32.4 [31.5, 33.4]	32.4 [31.7, 33.3]	32.2 [31.4, 33.2]	0.51
MCV,fL	92.2 [88.3, 96.5]	92.5 [89.0, 97.1]	93.0 [88.5, 96.6]	91.7 [88.3, 95.5]	91.5 [86.4, 96.9]	0.223
RDW,%	14.4 [13.5, 15.9]	14.4 [13.3, 15.9]	14.5 [13.5, 16.1]	14.5 [13.6, 15.9]	14.4 [13.7, 15.9]	0.695
INR,s	1.3 [1.2, 1.6]	1.3 [1.2, 1.6]	1.3 [1.2, 1.6]	1.3 [1.2, 1.5]	1.3 [1.1, 1.5]	0.078
PT,s	14.4 [12.8, 17.0]	14.4 [12.6, 18.0]	14.5 [13.0, 16.9]	14.3 [12.8, 16.5]	13.6 [12.2, 15.7]	0.057
PTT,s	32.3 [27.9, 47.1]	31.7 [28.1, 47.2]	32.6 [28.3, 46.6]	32.5 [27.4, 47.8]	32.2 [27.2, 44.9]	0.947
Aniongap,mmol/L	14.0 [11.7, 16.7]	13.4 [11.0, 16.0]	14.0 [12.0, 17.0]	14.0 [11.5, 16.6]	15.0 [12.8, 18.5]	**0.001**
Bicarbonate,mmol/L	22.0 [19.5, 24.3]	22.3 [20.4, 24.4]	22.0 [20.0, 24.2]	22.0 [19.2, 24.4]	20.5 [17.9, 24.0]	**0.008**
BUN,mg/dL	23.8 [15.5, 39.3]	20.0 [14.0, 32.4]	23.0 [15.5, 39.0]	27.0 [17.0, 43.8]	33.2 [23.1, 54.8]	** < 0.001**
Calcium,mg/dL	8.5 [8.0, 8.8]	8.6 [8.1, 9.0]	8.4 [8.0, 8.8]	8.4 [8.0, 8.7]	8.2 [7.7, 8.6]	** < 0.001**
Chloride,mEq/L	103.2 [99.3, 106.7]	103.5 [100.0, 107.0]	103.0 [99.1, 106.7]	103.3 [99.5, 106.1]	102.7 [97.3, 105.0]	0.092
Creatinine,mg/dL	1.2 [0.9, 1.9]	1.0 [0.8, 1.6]	1.2 [0.9, 1.8]	1.2 [0.9, 2.1]	1.7 [1.1, 2.9]	** < 0.001**
Potassium,mEq/L	4.2 [3.9, 4.6]	4.1 [3.8, 4.5]	4.2 [3.8, 4.5]	4.3 [3.9, 4.6]	4.5 [4.1, 5.0]	** < 0.001**
Sodium,mEq/L	139.0 [135.8, 142.0]	139.3 [136.0, 142.0]	139.0 [135.6, 141.7]	139.0 [136.0, 142.0]	138.0 [135.3, 140.7]	0.204
TyG index,mg/dL	9.0 [8.5, 9.5]	8.4 [8.2, 8.5]	9.0 [8.8, 9.1]	9.5 [9.4, 9.7]	10.3 [10.1, 10.4]	** < 0.001**
LOS in ICU,Day	7.8 [5.0, 13.6]	6.0 [4.3, 10.0]	7.8 [4.9, 12.9]	10.1 [6.2, 18.2]	12.6 [7.0, 23.4]	** < 0.001**
30-day mortality,N (%)	319 (28.8)	88 (24.2)	110 (28.4)	77 (31.2)	44 (40.0)	**0.011**
90-day mortality,N (%)	411 (37.1)	118 (32.4)	145 (37.5)	94 (38.1)	54 (49.1)	**0.016**
180-day mortality,N(%)	459 (41.4)	136 (37.4)	164 (42.4)	104 (42.1)	55 (50.0)	0.112
365-day mortality,N(%)	542 (48.9)	168 (46.2)	190 (49.1)	120 (48.6)	64 (58.2)	0.179

Continuous variables are presented as mean ± SD if normally distributed, and median (interquartile range) if not normally distributed. Categorical variables are presented as number of patients (%). SBP, systolic blood pressure; DBP, diastolic blood pressure; MBP, mean blood pressure; HR, heart rate; RR, respiratory rate; Spo2, oxygen saturation; SOFA, sequential organ failure assessment; APSIII, acute physiology score III; GCS, Glasgow coma scale; OASIS, oxford acute severity of illness score; SAPS II, simplified acute physiology score; BUN, blood urea nitrogen; INR, International Normalized Ratio; PT, prothrombin time; PTT, partial prothrombin time; WBC, white blood cell; MCH, mean corpuscular hemoglobin; MCHC, mean corpuscular hemoglobin concentration; MCV, mean corpuscular volume; RBC, red blood cell; RDW, red cell distribution width; TyG, triglyceride glucose; LOS, length of stay; ICU, intensive care unit. The P-values with statistical significance are bolded

Moreover, the LOS in ICU, the 30-day mortality, the 90-day mortality, the 180-day mortality and the 365-day mortality in traj1 group were lower than those in other groups. Patients in traj4 group with the highest average TyG index level were younger, exhibited the lowest prevalence of cerebrovascular disease and the highest percentage of the 30-day, 90-day, 180-day and 365-day mortality.

### Survival analysis

**Fig. 3:**
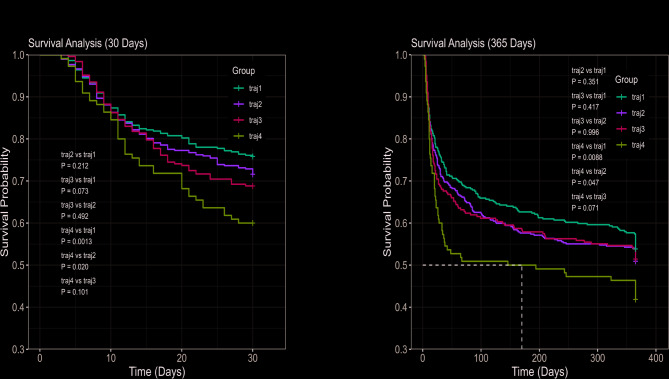
Kaplan–Meier survival analysis for all-cause mortality among each triglyceride-glucose (TyG) index trajectory. Kaplan–Meier curves of 30-day (**A**) and 365-day (**B**) all-cause mortality stratified by TyG index trajectories. *Note*: traj1, stable-low group; traj2, slowly ascend group; traj3, ascend-descend group; traj4, fluctuate-high group

Kaplan–Meier survival analysis revealed the differences of the 30-day, 90-day, 180-day and 365-day mortality between different TyG index trajectory groups. The 30-day mortality rate progressively increased from group traj1 to traj4, with statistically significant differences observed between groups (*p* < 0.05). This suggests a linear correlation between rising TyG index values and increasing mortality rates. When comparing outcomes across different timepoints, all trajectory groups demonstrated rising mortality trends at 90-day, 180-day and 365-day follow-ups compared to 30-day outcomes. These findings indicate that among critically ill ICU patients, mortality rates show time-dependent escalation with prolonged observation periods. In detail, AF patients in the traj4 group showed the highest 30-day (24.2%, 28.4%, 31.2%, 40.0%, traj1 vs. traj4: *p* = 0.0013, traj2 vs. traj4: *p* = 0.020), 90-day (32.4%, 37.5%, 38.1%, 49.1%, traj1 vs. traj4: *p* < 0.001, traj2 vs. traj4: *p* = 0.0018, traj3 vs. traj4: *p* = 0.047), 180-day (37.4%, 42.4%, 42.1%, 50.0%, traj1 vs. traj4: *p* = 0.0072) and 365-day (46.2%, 49.1%, 48.6%, 58.2%, traj1 vs. traj4: *p* = 0.0088, traj2 vs. traj4: *p* = 0.047) mortality rate compared to patients in other trajectory groups. The result of log-rank tests between different groups were shown in Fig. 3.

### Relationships between TyG index trajectory and clinical outcomes of patients with AF

**Fig. 4:**
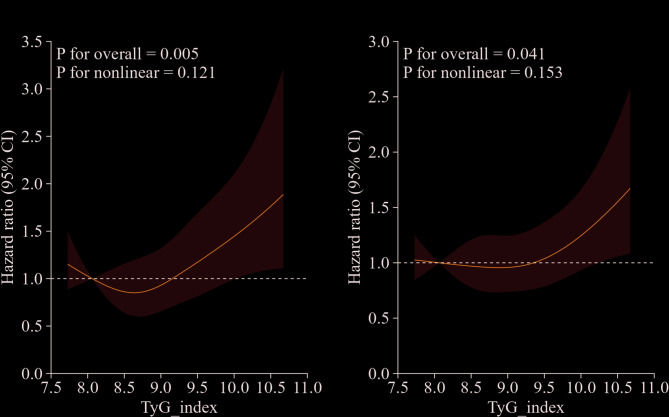
Restricted cubic spline analysis of TyG index with 30-day (**A**) and 365-day (**B**) all-cause mortality

In the univariate analysis, we selected potential confounding covariates based on predefined criteria for adjustment in the multivariable Cox proportional hazards models. The detailed results are provided in the supplementary materials. In order to determine the independent effects of TyG index trajectory on mortality, three Multivariable Cox proportional hazard models were applied (Table 3). Crude model is unadjusted by any confounders. Model I adjusted for age, gender, race, heart rate, weight. Model II adjusted for amiodarone, congestive heart failure, liver disease, diabetes, statin, sepsis, hypertension, beta-blocker, WBC, RBC, BUN, RDW, creatinine and all variables in Model I. In the Crude model, compared to the reference group (traj1), the 30-day, 90-day, 180-day and 365-day mortality risk of traj4 group were 1.79 (95% CI 1.24–2.57, *p* = 0.002), 1.71 (95% CI 1.24–2.35, *p* = 0.001), 1.53 (95% CI 1.12–2.09, *p* < 0.01), 1.46 (95% CI 1.09–1.94, *p* = 0.010) respectively. In the Model I, the 30-day, 90-day, 180-day and 365-day mortality risk of traj4 group compared to the reference group were 2.35 (95% CI 1.61–3.45, *p* < 0.001), 2.29 (95% CI 1.63–3.22, *p* < 0.001), 2.05 (95% CI 1.47–2.86, *p* < 0.001), 2.00 (95% CI 1.47–2.71, *p* < 0.001). In the Model II, the traj4 group showed a similar trend of the 30-day, 90-day, 180-day and 365-day mortality risk compared to traj1. The results were 1.71 (95% CI 1.14–2.56, *p* < 0.01), 1.67 (95% CI 1.17–2.39, *p* < 0.01), 1.44 (95% CI 1.03–2.06, *p* < 0.05), 1.44 (95% CI 1.04–1.98, *p* < 0.05) respectively. Compared with traj1 group, patients in traj2 and traj3 groups showed no difference in 30-day, 90-day, 180-day or 365-day all-cause mortality rates. These results indicated that patients in traj4 group exhibited higher mortality risk both in short-term and long-term follow up. Additionally, the RCS revealed an increasing trend in all-cause mortality with higher TyG index levels, showing statistically significant linear associations at 30 days (P for nonlinearity = 0.121, P for overall = 0.005) and 365 days (P for nonlinearity = 0.153, P for overall = 0.041) (Fig. 4).

**Table 3:** Hazard ratios (95% confidence intervals) of 30-day and 365-day mortality risk in patients with atrial fibrillation by trajectory groups of triglyceride-glucose index

	Crude Model		Model I		Model II	
	Crude HR (95%CI)	P-value	Adjusted HR (95%CI)	P-value	Adjusted HR (95%CI)	P-value
*30-day mortality*						
traj1	Reference		Reference		Reference	
traj2	1.19 [0.90, 1.57]	0.229	1.24 [0.93, 1.64]	0.137	1.09 [0.81, 1.45]	0.572
traj3	1.30 [0.96, 1.77]	0.094	1.54 [1.12, 2.11]	** < 0.01**	1.28 [0.92, 1.79]	0.142
traj4	1.79 [1.24, 2.57]	**0.002**	2.35 [1.61, 3.45]	** < 0.001**	1.71 [1.14, 2.56]	** < 0.01**
*365-day mortality*						
traj1	Reference		Reference		Reference	
traj2	1.09 [0.89, 1.35]	0.396	1.16 [0.96, 1.43]	0.171	0.99 [0.80, 1.23]	0.957
traj3	1.09 [0.86, 1.37]	0.489	1.31 [1.03, 1.67]	** < 0.05**	1.08 [0.84, 1.39]	0.556
traj4	1.46 [1.09, 1.94]	**0.010**	2.00 [1.47, 2.71]	** < 0.001**	1.44 [1.04, 1.98]	** < 0.05**

Note: traj1, stable-low group; traj2, slowly ascend group; traj3, ascend-descend group; traj4, fluctuate-high group;P-values with statistical significance are bolded

**Crude Model**: unadjusted; **Model I**: adjusted for Age, Gender, Race, Heart rate, Weight; **Model II**: adjusted for Amiodarone, Congestive heart failure, Liver disease, Diabetes, Statin, Sepsis, Hypertension, Beta-blocker, WBC, RBC, BUN, RDW, Creatinine and all variables in Model I;

HR, hazard ratio; CI, confidence interval; WBC, white blood cell; RBC, red blood cell; BUN, blood urea nitrogen; RDW, red cell distribution width;

### Subgroup analysis

**Fig. 5:**
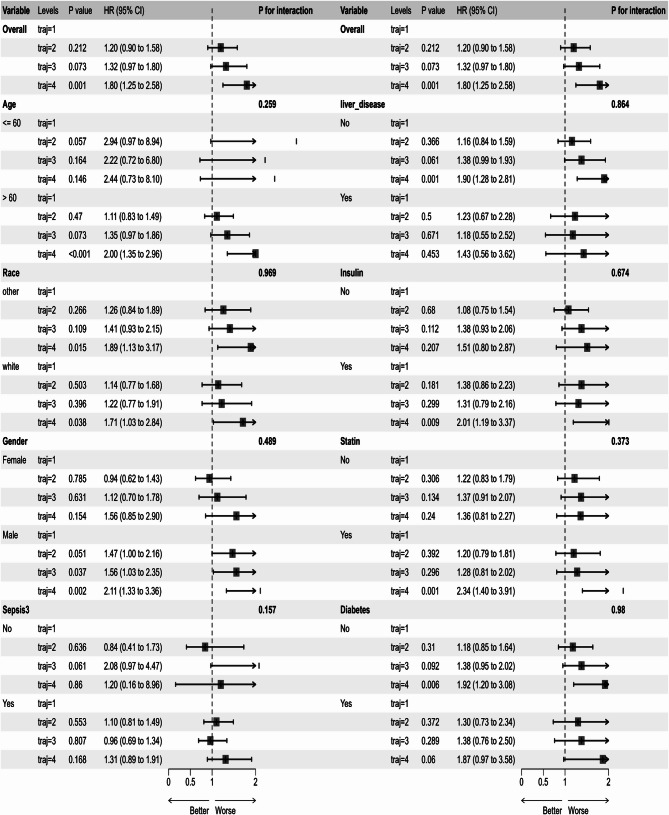
Subgroup analysis of the associations between TyG index trajectories and 30-day all-cause mortality. *Note*: traj1, stable-low group; traj2, slowly ascend group; traj3, ascend-descend group; traj4, fluctuate-high group

**Fig. 6:**
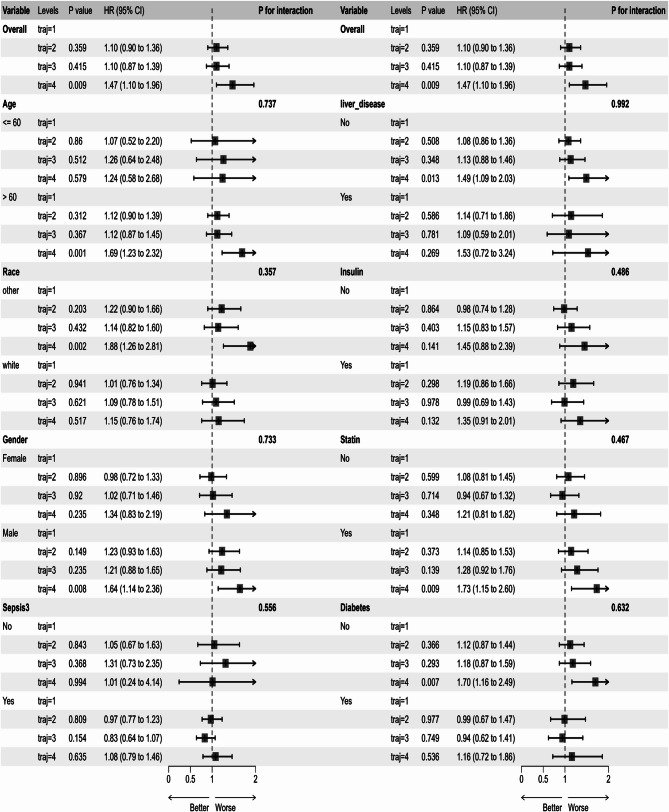
Subgroup analysis of the associations between TyG index trajectories and 365-day all-cause mortality. *Note*: traj1, stable-low group; traj2, slowly ascend group; traj3, ascend-descend group; traj4, fluctuate-high group

To further evaluate the robustness of the relationships between TyG index trajectories and 30-day, 90-day, 180-day and 365-day mortality in various conditions, we tested cross-interactions between TyG index trajectories and age, gender, race, sepsis, liver disease, usage of insulin, usage of statin and diabetes. To minimize potential false-positive findings, we implemented Bonferroni correction for multiple testing adjustment. In our study, no interactions were found (P for interaction > 0.05), indicating robustness of the outcomes, as shown in Figs. 5 and 6. In subgroups of patients aged ≥ 60 years, male, without liver disease, using statin and without diabetes, the HR for 30-day and 365-day mortality showed significant difference (*p* < 0.05) in traj4 group compared to traj1 group. Whereas, in patients with or without sepsis, the HRs of 30-day, 90-day, 180-day and 365-day mortality showed no statistical significance among different groups. Interestingly, only the 30-day and 90-day HRs in patients with using insulin were statistically significant (*p* < 0.05) in traj4. Regardless of whether the patient was White or of another race, the 30-day HR showed statistical significance. However, in other races, the 90-day, 180-day, and 365-day HRs were also statistically significant. Notably, in our subgroup analysis, statin use and non-diabetic status in traj4 showed significantly stronger associations with all-cause mortality compared to other groups.

## Discussion

In our retrospective observational study, we used GBTM to identify four TyG index patterns in critically ill patients with AF in ICU. As far as we known, our study is the first retrospective investigation to explore the correlation between TyG index trajectories and all-cause mortality in critically ill patients with AF stayed in ICU. Those patients with higher and unstable TyG index were association with a higher risk of 30-day,90-day,180-day and 365-day mortality compared with patients whose TyG index sustained at a lower level. This association still remained the same after adjusting potential influencing factors. These results indicated that different TyG index trajectory models can help identify patients with poorer clinical outcomes and provide clinical guidelines for ICU management.

Growing evidence demonstrated that IR and its relevant metabolic disorders contributed to various cardiovascular diseases (CVD) [5, 26–30]. Lots of researches have reported that IR is a significant risk factor for the development and progression of AF. The main reason owed to left atrial fibrosis and structural remodeling [16, 31]. Inflammation or myocardial steatosis can lead to both structural and electrical remodeling of the left atrium. The accumulation of intra-cellular triglycerides elevates free fatty acid levels and promotes the formation of toxic lipids which accelerates myocardial apoptosis and fibrosis. The combination of atrial fibrosis and retrograde pressure secondary to left ventricular diastolic dysfunction leads to atrial dilatation, which in turn drives structural and electrical remodeling of the left atrium [32]. At the cellular level, structural and electrical remodeling promotes excessive calcium release from the sarcoplasmic reticulum, thereby facilitating atrial fibrillation triggers [32, 33]. In addition, structural and functional remodeling of cardiac sympathetic innervation appears to be an important driver of AF development and maintenance [34]. Thus, IR is one of the key factors in the development and progression of AF, particularly in the ICU setting, where it is often accompanied by stress-induced hyperglycemia and exacerbated IR. The impact of IR on AF patients in the ICU warrants further investigation. However, current methods for assessing IR in critically ill patients are often expensive and time-consuming.

Fortunately, TyG index is a good surrogate maker for IR, with more and more researches confirming that TyG index is strongly associated with CVD and has the same association in critically ill patients [16, 35–40]. The TyG index has emerged as a simple yet effective prognostic tool for patients with atrial fibrillation AF, regardless of ICU admission status. A meta-analysis including 6 studies with 5,813 patients revealed that the TyG index is significantly associated with atrial fibrillation AF risk [9]. In a community-based cohort study of 1,979 Chinese AF patients with median 5.31-year follow-up, higher TyG index quartiles showed significantly increased risk of major adverse cardiovascular and cerebrovascular events (MACCE) compared to lower quartiles (HR = 2.041 95% CI 1.391–2.994, *p* < 0.001) [41]. In a retrospective cohort study included 864 AF patients without diabetes, researchers observed that higher TyG index correlates with poor cardiovascular prognosis in this population of patients[42]. Another retrospective study of 2,509 critically ill AF patients demonstrated that those with higher TyG-body mass index (BMI) index levels had significantly greater mortality risk at 30, 90, 180 and 365 days after ICU admission compared to patients with lower levels [16]. Current researches consistently demonstrate that elevated TyG index values are associated with worse clinical outcomes in AF patients.

However, existing studies predominantly rely on single time point assessments, which fail to capture the temporal fluctuation inherent of this index. These static analytical models provide incomplete representations of patients’ longitudinal trajectories, thereby limiting their prognostic value and clinical application. To address this critical gap, we urgently need innovative dynamic modeling frameworks that can both identify early-stage deterioration trends and facilitate real-time treatment adjustments.

Therefore, dynamic trajectory may be used to fill this gap. Rely on the dynamic trajectory, we can adequately consider the association from a whole timeline. Previous studies have shown that TyG index trajectory was relevant with some CVD. Hong et al. conducted a retrospective cohort study revealed that TyG index trajectories were independently associated with MACE in elderly heart failure patients with diabetes [43]. Feng et al. found that baseline TyG index levels and high growth of TyG index trajectories were associated with the occurrence of hypertension [44]. Another prospective and multi-center cohort study with a longer follow-up indicated persistently elevated TyG index in patients with hypertension was associated with an increased risk of stroke, particularly ischemic stroke [45]. Besides, researchers found that different TyG index trajectories were strongly associated with the AF occurrence in patients who had undergone successful percutaneous or surgical AF ablation. They suggested this association might be mediated through insulin resistance’s effects on atrial electrical remodeling [46]. Although numerous studies have established the relationship between the TyG index and AF, research investigating the association between dynamic TyG index trajectories and all-cause mortality in critically ill AF patients remains lacking.

In our study, based on GBTM we generated 4 groups of TyG index trajectory models. We observed that TyG index fluctuated at high levels could be regarded as an independent mortality risk factor for AF patients in ICU. This association remained significant after adjusting relevant covariables. Moreover, compared to groups with a consistently high TyG index, the lowest-level group did not show a significantly elevated mortality risk. This suggested that maintaining a stable TyG index over time may be associated with a lower mortality risk compared to experiencing fluctuation at the same average level. The TyG index, which is based on glucose and triglyceride levels, may fluctuate due to changes in either parameter. Typically, glucose levels are the primary contributor to these fluctuations. These fluctuations reflect alterations in insulin resistance and are associated with an increased risk of CVD. The underlying mechanism may involve glucose and triglyceride variability, which can activate oxidative stress and promote the production of inflammatory cytokines, leading to endothelial damage and dysfunction. In a foundational study, researchers found that intermittent exposure to a high-glucose environment increased reactive oxygen species (ROS) release in endothelial cells and induced apoptosis compared to normal and low glucose levels in rats. This effect occurs because ROS stimulate the expression of TNF-α, a pro-apoptotic factor, which in turn further promotes ROS production, creating a vicious cycle. These findings suggest that glucose fluctuations contribute to cardiac fibrosis and AF [47]. Other preclinical studies have also demonstrated that glucose variability can increase susceptibility to arrhythmias by influencing ROS levels. Additionally, it may exacerbate cardiac fibrosis by inhibiting the AKT signaling pathway [48, 49]. Additionally, hyperglycemia and increased glucose variability may contribute to AF by mediating abnormal autonomic activity [50, 51]. These potential mechanisms suggest that blood glucose fluctuation is more likely to trigger inflammatory factor release than stable glucose levels. Such fluctuation exacerbate oxidative free radical damage and activate fibrosis pathways. In critically ill patients, repeated glucose variability may aggravate myocardial fibrosis progression, further impairing cardiac function and potentially increasing mortality risk.

Our study suggested that TyG index variability in critically ill AF patients requires greater clinical attention and intervention. Furthermore, we identified age, BUN, SOFA score and sepsis as independent risk factors, which aligns with previous studies [17, 52, 53]. In survival analysis, the traj4 group exhibited significantly lower survival times compared to other groups, both in short-term or long-term time follow-up periods (Fig. 3). Using restricted cubic spline (RCS) analysis, we also identified a linear dose–response relationship between the elevation of TyG index levels and adverse outcomes (Fig. 4). This suggests that AF patients with persistently elevated TyG index levels are associated with higher risk of excess mortality. In subgroup analyses, critically ill AF patients with elevated and fluctuating TyG index levels demonstrated significantly higher all-cause mortality across all follow-up periods (30-day, 90-day, 180-day and 365-day). This association was particularly prominent in male, elderly patients (> 60 years), statin users, and those without liver disease or diabetes. These results in our study were in consistent with previous literature [16, 54]. Based on our subgroup analysis, we believe the prominent effect observed in statin users and non-diabetes patients deserves further explanation. In the non-diabetic population, patients tend not to use insulin or oral hypoglycemic medications before ICU admission. However, some may have un-diagnosed diabetes but were not included in the diabetic group due to low detection rates, which could influence our results. Additionally, in the diabetic population, intensive insulin therapy is commonly administered in the ICU, and this approach is associated with reduced mortality [55, 56]. Statin use may increase diabetes incidence, worsen glycemic control, and elevate the risk of MACE [57]. This is particularly relevant in ICU settings where higher-risk patients are more likely to receive statin therapy. The glucose-elevating effects of statin may contribute to their inclusion in the TyG fluctuation group, potentially explaining why statin users in this group showed higher risk and poor prognosis. However, current evidence regarding the underlying mechanisms remains insufficient. Further studies are needed to elucidate these interactions.

As an easily accessible and serially measurable biomarker, comprehensive TyG index trajectory monitoring enables clinicians to: (1) promptly identify high-risk AF patients, (2) optimize treatment strategies, and (3) ultimately reduce mortality while improving clinical outcomes. In the ICU, patients routinely require regular laboratory monitoring, making the TyG index trajectory both accessible and cost-effective without incurring additional expenses. Furthermore, when identifying an AF patient with an elevated TyG index that fluctuates over time, we can implement more intensive interventions to reduce insulin resistance and stabilize metabolic parameters particularly in non-diabetic patients. Additionally, patients should receive dietary guidance and medication instructions for post-ICU management.

This study explore the association between TyG index trajectories and all-cause mortality in ICU patients with AF. The most strength of the present study is that we use GBTM to identify different TyG index trajectories in ICU populations with AF. This advanced approach can help us identify subgroups in a population with similar patterns of change over time. It ensure us to identify subgroups within a population that present similar patterns of change in the TyG index over time. By distinguishing among these trajectories, we gained more understanding of how different change of TyG index over time influence all-cause mortality following ICU admission in AF patients.

However, there are several limitations in our study. Firstly, as a retrospective study we can’t verify a causal relationship between TyG index trajectories and mortality. Further prospective study and randomized controlled trial are needed to validate the relationship. Secondly, our study is a single-center study which was based on the observational data extracted from MIMIC-IV database. The sample size of our study was moderate and this study only included patients in the USA, which may limit our result extrapolating to other countries. Multi-center and large sample size cohort study is needed, in our next research we will add other databases or hospital data from real world. Thirdly, due to the particularity in ICU, we can’t guarantee the glucose and triglyceride all were obtained from fasting patients. However, based on previously published studies conducted under similar conditions, the TyG index has demonstrated significant predictive capacity for clinical outcomes. Although triglycerides levels are not routinely repeatedly measured during ICU stays, the TyG index is calculated using both blood glucose and triglyceride data. Given that triglyceride levels tend to remain relatively stable over short periods, changes in glucose levels can serve as a proxy for the dynamic changes in the TyG index. In comparison to other studies, we try our best to match different glucose with triglyceride data within a 24-h time window and have included only patients with at least three records of either glucose or triglycerides in our study. These approaches enhance the reliability of our finding. Finally, since our data were derived from exist database, it is important to acknowledge that the potential influence of unmeasured confounders on our findings cannot be entirely eliminated, despite our adjustment for well-known confounders. Addressing these limitations in future studies could provide a more comprehensive understanding of the TyG trajectory’s role in predicting outcomes among critically ill AF patients [14–18, 58].

## Conclusions

Our result revealed the fluctuation of TyG index in high level in critically ill AF patients is associated with increased mortality at 30-day, 90-day, 180-day and 365-day. To our knowledge, our study is the first to explore the association between TyG index trajectories and all-cause mortality in ICU patients with AF. As an easily obtainable and repeatedly measurable index, comprehensive TyG index trajectory monitoring can effectively guide clinicians to promptly identify high-risk AF patients, select optimal treatment strategies and ultimately reduce mortality while improving patient’s prognosis. As a strong indicator for risk stratification and prognosis assessment, the association between TyG index trajectories and prognosis in critically ill patients with AF deserve further study.

## Electronic supplementary material

Below is the link to the electronic supplementary material.

Supplementary Material

## Data Availability

Publicly available datasets were analyzed in this study. This data can be download here: https://mimic.mit.edu/.

## Abbreviations

TyG: Triglyceride glucose
AF: Atrial fibrillation
MIMIC: Medical Information Mart for Intensive Care
ICU: Intensive care unit
GBTM: Group-based trajectory models
HRs: Hazard ratios
CI: Confidence interval
RCS: Restricted cubic splines
LOS: Length of stay
AIC: Akaike information Criterion
BIC: Bayesian information Criteria
Avepp: Average posterior probabilities
BUN: Blood urea nitrogen
T2DM: Type 2 diabetes mellitus
CVD: Cardiovascular disease
BMI: Body mass index
MACE: Major adverse cardiovascular events
