# Supplementary material for: Association between trajectory of triglyceride-glucose index and all-cause mortality in critically ill patients with atrial fibrillation: a retrospective cohort study
Source: Cardiovasc Diabetol. 2025 Jul 10;24:278. doi: 10.1186/s12933-025-02838-x (PMC12243274; doi:10.1186/s12933-025-02838-x)
Supplement: Supplementary file 1 — Supplementary Material [file 12933_2025_2838_MOESM1_ESM.docx]

**Association between trajectory of triglyceride-glucose index and all-cause mortality in critically ill patients with Atrial fibrillation: a retrospective cohort study**

**Supplementary materials**

**Supplementary Table 1.** Missing variables and the situation of treatment

**Supplementary Table 2.** Univariate analysis at 365-day mortality

**Supplementary Table 3.** Hazard ratios (95% confidence intervals) of 90-day and 180-day mortality risk in patients with atrial fibrillation by trajectory groups of triglyceride-glucose index

**Supplementary Fig.1** the figures of Schoenfeld residual tests

**Supplementary Fig.2** Subgroup analysis of the associations between TyG index trajectories and 90-day all-cause mortality.

**Supplementary Fig.3** Subgroup analysis of the associations between TyG index trajectories and 180-day all-cause mortality.

**Supplementary Fig.4** Kaplan–Meier survival analysis for all-cause mortality at 90-day among each triglyceride-glucose (TyG) index trajectory.

**Supplementary Fig.5**  Kaplan–Meier survival analysis for all-cause mortality at 180-day among each triglyceride-glucose (TyG) index trajectory.

**Part relevant code**

**Supplementary table.1 Missing variables and the situation of treatment**

| **Missing variables** | **Missing value** | **treatment** |
| --- | --- | --- |
| RBC | 1.5% | imputation |
| WBC | 1.4% | imputation |
| hematocrit | 1.4% | imputation |
| hemoglobin | 1.5% | imputation |
| platelet | 1.6% | imputation |
| MCH | 1.5% | imputation |
| MCHC | 1.5% | imputation |
| MCV | 1.5% | imputation |
| RDW | 1.6% | imputation |
| PTT | 5.2% | deletion |
| height | 26.8% | deletion |
| Anion gap | 1.6% | imputation |
| bicarbonate | 1.3% | imputation |
| BUN | 1.3% | imputation |
| calcium | 5.3% | imputation |
| chloride | 1.3% | imputation |
| creatinine | 1.3% | imputation |
| potassium | 1.3% | imputation |
| sodium | 1.3% | imputation |
| lymphocytes | 52.3% | deletion |
| neutrophils | 52.3% | deletion |
| weight | 2.7% | deletion |

BUN, blood urea nitrogen; INR, International Normalized Ratio; PTT, partial prothrombin time; WBC, white blood cell; MCH, mean corpuscular hemoglobin; MCHC, mean corpuscular hemoglobin concentration; MCV, mean corpuscular volume; RBC, red blood cell; RDW, red cell distribution width;

**Supplementary table.2 Univariate analysis at 365-day mortality**

| **Variable** | **n_contrast** | **n_ref** | **beta** | **HR** | **lower_95** | **upper_95** | **p.value** | **global.pval** |
| --- | --- | --- | --- | --- | --- | --- | --- | --- |
| acei | 43 | 1065 | -0.761 | 0.467 | 0.264 | 0.828 | 0.01 | 0.003 |
| amiodarone | 219 | 889 | 0.281 | 1.32 | 1.08 | 1.62 | 0.01 | 0.007 |
| aspirin | 565 | 543 | -0.335 | 0.716 | 0.604 | 0.847 | 0.00 | 0.0001 |
| Beta_blocker | 374 | 734 | 0.0345 | 1.04 | 0.867 | 1.24 | 0.70 | 0.703 |
| clopidogrel | 97 | 1011 | 0.101 | 1.11 | 0.829 | 1.48 | 0.49 | 0.5 |
| digoxin | 47 | 1061 | 0.356 | 1.43 | 0.988 | 2.06 | 0.06 | 0.072 |
| myocardial_infarct | 282 | 826 | 0.144 | 1.16 | 0.958 | 1.39 | 0.13 | 0.136 |
| congestive_heart_failure | 540 | 568 | 0.13 | 1.14 | 0.962 | 1.35 | 0.13 | 0.13 |
| peripheral_vascular_disease | 160 | 948 | 0.0681 | 1.07 | 0.846 | 1.35 | 0.57 | 0.573 |
| cerebrovascular_disease | 502 | 606 | 0.0416 | 0.959 | 0.81 | 1.14 | 0.63 | 0.63 |
| chronic_pulmonary_disease | 238 | 870 | 0.211 | 1.23 | 1.01 | 1.5 | 0.04 | 0.0398 |
| rheumatic_disease | 40 | 1068 | 0.319 | 1.38 | 0.913 | 2.07 | 0.13 | 0.146 |
| liver_disease | 129 | 979 | 0.684 | 1.98 | 1.58 | 2.48 | <0.001 | <0.001 |
| diabetes | 392 | 716 | 0.0508 | 1.05 | 0.883 | 1.25 | 0.57 | 0.57 |
| renal_disease | 324 | 784 | 0.309 | 1.36 | 1.14 | 1.63 | <0.001 | 0.007 |
| heparin | 444 | 664 | 0.159 | 1.17 | 0.989 | 1.39 | 0.07 | 0.0674 |
| statin | 624 | 484 | -0.297 | 0.743 | 0.628 | 0.879 | <0.001 | 0.000573 |
| sepsis | 785 | 323 | 0.88 | 2.41 | 1.93 | 3 | <0.001 | <0.001 |
| warfarin | 12 | 1096 | -0.307 | 0.736 | 0.305 | 1.77 | 0.49 | 0.472 |
| CCB | 236 | 872 | -0.241 | 0.786 | 0.633 | 0.975 | 0.03 | 0.0246 |
| hypertension | 383 | 725 | -0.18 | 0.835 | 0.696 | 1 | 0.05 | 0.0494 |
| insulin | 485 | 623 | 0.223 | 1.25 | 1.06 | 1.48 | 0.01 | 0.00957 |
| gender | 678 | 430 | -0.18 | 0.835 | 0.704 | 0.991 | 0.04 | 0.0396 |
| SOFA | 1108 | 1108 | 0.0873 | 1.09 | 1.07 | 1.11 | <0.001 | <0.001 |
| APSIII | 1108 | 1108 | 0.0158 | 1.02 | 1.01 | 1.02 | <0.001 | <0.001 |
| RBC | 1108 | 1108 | -0.312 | 0.732 | 0.655 | 0.817 | <0.001 | <0.001 |
| WBC | 1108 | 1108 | 0.0197 | 1.02 | 1.01 | 1.03 | <0.001 | 0.0003 |
| hematocrit | 1108 | 1108 | 0.0346 | 0.966 | 0.954 | 0.978 | <0.001 | <0.001 |
| hemoglobin | 1108 | 1108 | -0.123 | 0.885 | 0.851 | 0.919 | <0.001 | <0.001 |
| platelet | 1108 | 1108 | 0.000438 | 1 | 0.999 | 1 | 0.38 | 0.374 |
| MCH | 1108 | 1108 | 0.00978 | 0.99 | 0.96 | 1.02 | 0.53 | 0.532 |
| MCHC | 1108 | 1108 | -0.139 | 0.87 | 0.827 | 0.916 | <0.001 | <0.001 |
| MCV | 1108 | 1108 | 0.0149 | 1.02 | 1 | 1.03 | 0.01 | 0.0146 |
| RDW | 1108 | 1108 | 0.111 | 1.12 | 1.09 | 1.15 | <0.001 | <0.001 |
| INR | 1108 | 1108 | 0.304 | 1.35 | 1.25 | 1.47 | <0.001 | <0.001 |
| PT | 1108 | 1108 | 0.0326 | 1.03 | 1.03 | 1.04 | <0.001 | <0.001 |
| PTT | 1108 | 1108 | 0.00731 | 1.01 | 1 | 1.01 | <0.001 | <0.001 |
| GCS | 1108 | 1108 | 0.0194 | 0.981 | 0.945 | 1.02 | 0.31 | 0.323 |
| aniongap | 1108 | 1108 | 0.0644 | 1.07 | 1.05 | 1.08 | <0.001 | <0.001 |
| bicarbonate | 1108 | 1108 | -0.02 | 0.98 | 0.96 | 1 | 0.06 | 0.058 |
| BUN | 1108 | 1108 | 0.0141 | 1.01 | 1.01 | 1.02 | <0.001 | <0.001 |
| calcium | 1108 | 1108 | 0.0655 | 0.937 | 0.835 | 1.05 | 0.27 | 0.265 |
| chloride | 1108 | 1108 | 0.0155 | 0.985 | 0.971 | 0.998 | 0.03 | 0.0272 |
| creatinine | 1108 | 1108 | 0.118 | 1.13 | 1.08 | 1.17 | <0.001 | <0.001 |
| potassium | 1108 | 1108 | 0.158 | 1.17 | 1.02 | 1.34 | 0.02 | 0.0262 |
| sodium | 1108 | 1108 | 0.00786 | 1.01 | 0.993 | 1.02 | 0.32 | 0.315 |
| OASIS | 1108 | 1108 | 0.0342 | 1.03 | 1.02 | 1.05 | <0.001 | <0.001 |
| charlson_comorbidity_index | 1108 | 1108 | 0.11 | 1.12 | 1.08 | 1.15 | <0.001 | <0.001 |
| age | 1108 | 1108 | 0.0243 | 1.02 | 1.02 | 1.03 | <0.001 | <0.001 |
| SAPS II | 1108 | 1108 | 0.0241 | 1.02 | 1.02 | 1.03 | <0.001 | <0.001 |
| heart_rate | 1108 | 1108 | 0.00497 | 1 | 1 | 1.01 | 0.04 | 0.0446 |
| SBP | 1108 | 1108 | 0.0034 | 0.997 | 0.992 | 1 | 0.16 | 0.159 |
| DBP | 1108 | 1108 | 0.0152 | 0.985 | 0.978 | 0.992 | <0.001 | <0.001 |
| MBP | 1108 | 1108 | 0.0153 | 0.985 | 0.977 | 0.992 | <0.001 | <0.001 |
| RR | 1108 | 1108 | 0.048 | 1.05 | 1.03 | 1.07 | <0.001 | <0.001 |
| spo2 | 1108 | 1108 | 0.0262 | 0.974 | 0.937 | 1.01 | 0.19 | 0.197 |
| weight | 1108 | 1108 | 0.00342 | 0.997 | 0.993 | 1 | 0.03 | 0.0274 |
| race | 566 | 542 | 0.012 | 1.01 | 0.855 | 1.2 | 0.89 | 0.889 |

SBP, systolic blood pressure; DBP, diastolic blood pressure; MBP, mean blood pressure; RR, respiratory rate; Spo2, oxygen saturation; SOFA, sequential organ failure assessment; APSIII, acute physiology score III; GCS, Glasgow coma scale; OASIS, oxford acute severity of illness score; SAPS II, simplified acute physiology score; BUN, blood urea nitrogen; INR, International Normalized Ratio; PT, prothrombin time; PTT, partial prothrombin time; WBC, white blood cell; MCH, mean corpuscular hemoglobin; MCHC, mean corpuscular hemoglobin concentration; MCV, mean corpuscular volume; RBC, red blood cell; RDW, red cell distribution width;

**Supplementary table.3** Hazard ratios (95% confidence intervals) of 90-day and 180-day mortality risk in patients with atrial fibrillation by trajectory groups of triglyceride-glucose index

|  |  | **Crude Model** |  |  |  | **Model I** |  |  |  | **Model II** |  |  |
| --- | --- | --- | --- | --- | --- | --- | --- | --- | --- | --- | --- | --- |
|  |  | **Crude HR (95%CI)** |  | **P-value** |  | **Adjusted HR (95%CI)** |  | **P-value** |  | **Adjusted HR (95%CI)** |  | **P-value** |
| 90-day mortality |  |  |  |  |  |  |  |  |  |  |  |  |
| traj1 |  | Reference |  |  |  | Reference |  |  |  | Reference |  |  |
| traj2 |  | 1.18 [0.92, 1.50] |  | 0.192 |  | 1.23 [0.96, 1.57] |  | 0.100 |  | 1.06 [0.83, 1.36] |  | 0.644 |
| traj3 |  | 1.20 [0.92, 1.58] |  | 0.185 |  | 1.42 [1.07, 1.88] |  | **<0.05** |  | 1.16 [0.87, 1.56] |  | 0.311 |
| traj4 |  | 1.71 [1.24, 2.35] |  | **0.001** |  | 2.29 [1.63, 3.22] |  | **<0.001** |  | 1.67 [1.17, 2.39] |  | **<0.01** |
| 180-day mortality |  |  |  |  |  |  |  |  |  |  |  |  |
| traj1 |  | Reference |  |  |  | Reference |  |  |  | Reference |  |  |
| traj2 |  | 1.16 [0.92, 1.46] |  | 0.202 |  | 1.22 [0.97, 1.53] |  | 0.095 |  | 1.04 [0.82, 1.31] |  | 0.749 |
| traj3 |  | 1.16 [0.89, 1.49] |  | 0.270 |  | 1.37 [1.06, 1.79] |  | **<0.05** |  | 1.11 [0.84, 1.47] |  | 0.450 |
| traj4 |  | 1.53 [1.12, 2.09] |  | **<0.01** |  | 2.05 [1.47, 2.86] |  | **<0.001** |  | 1.44 [1.03, 2.06] |  | **<0.05** |

Note: traj1, stable-low group; traj2, slowly ascend group; traj3, ascend-descend group; traj4, fluctuate-high group;

**Crude Model**: unadjusted; **Model Ⅰ**: adjusted for Age, Gender, Race, Heart rate, Weight; **Model Ⅱ**: adjusted for Amiodarone, Congestive heart failure, Liver disease, Diabetes, Statin, Sepsis3, Hypertension, Beta-blocker, WBC, RBC, BUN, RDW, Creatinine and all variables in Model Ⅰ;

HR, hazard ratio; CI, confidence interval; WBC, white blood cell; RBC, red blood cell; BUN, blood urea nitrogen; RDW, red cell distribution width;

**Supplementary Fig.1 the figures of Schoenfeld residual tests**


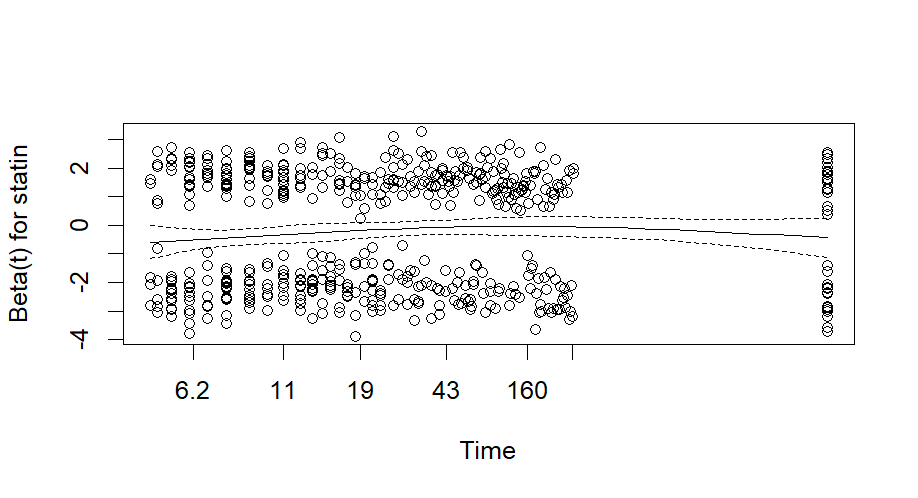


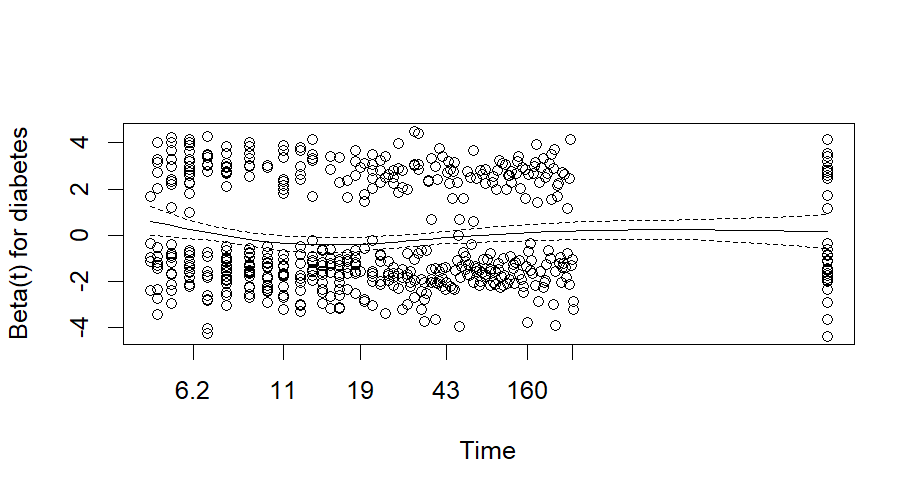


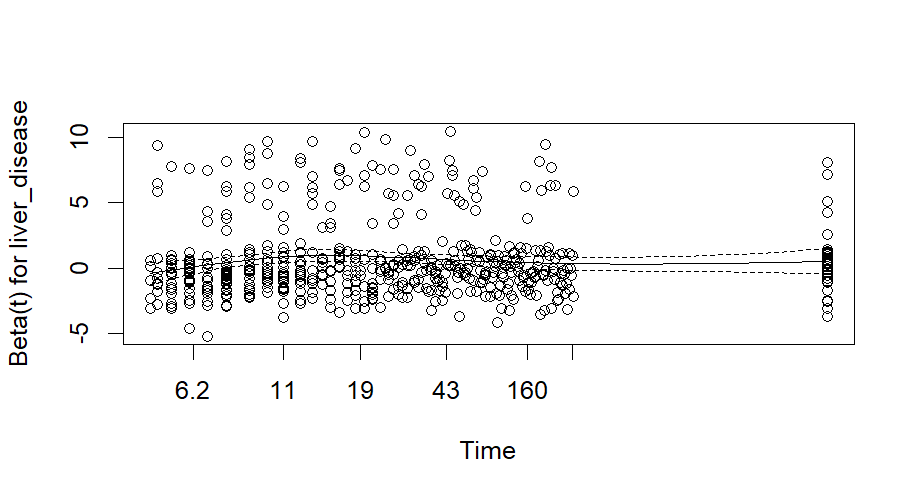


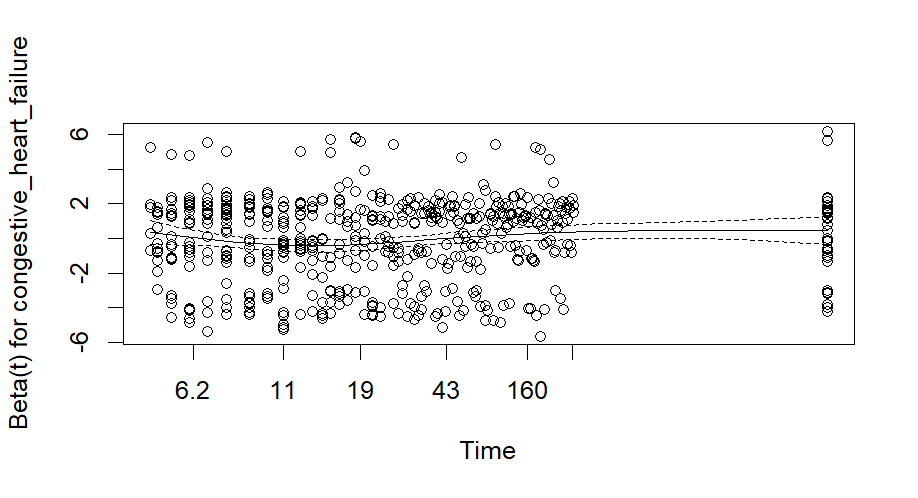


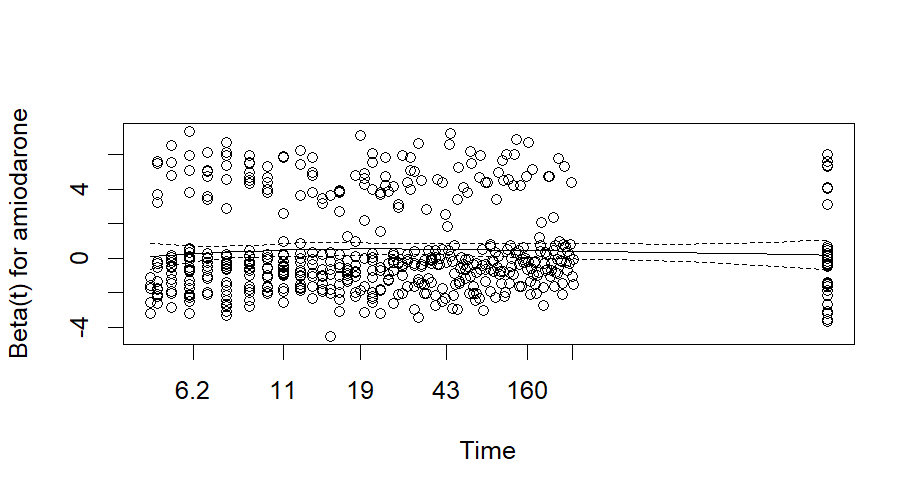


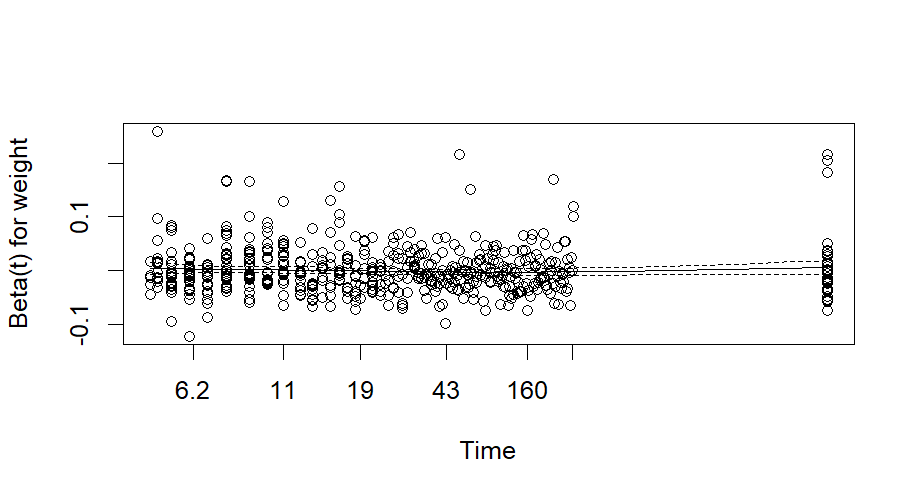

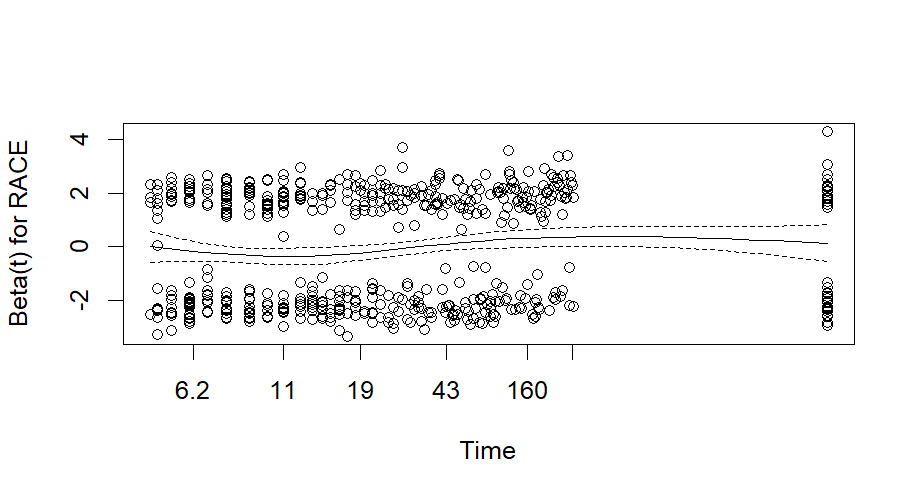

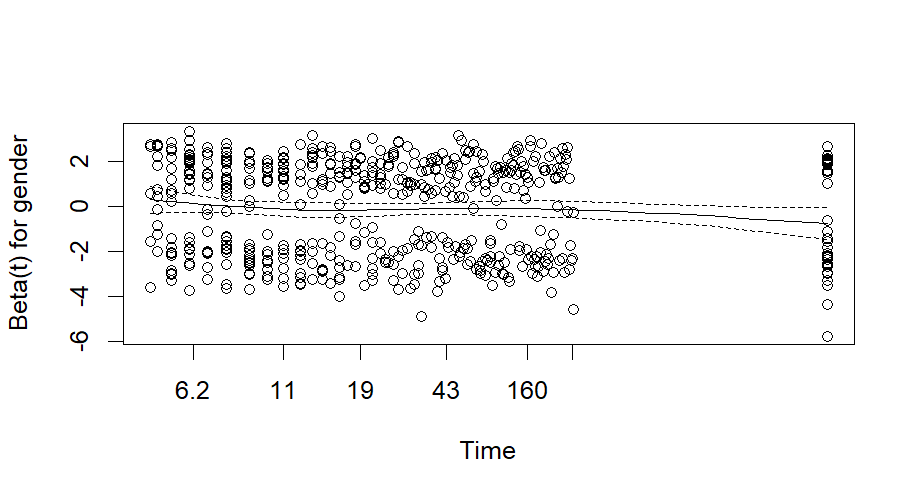

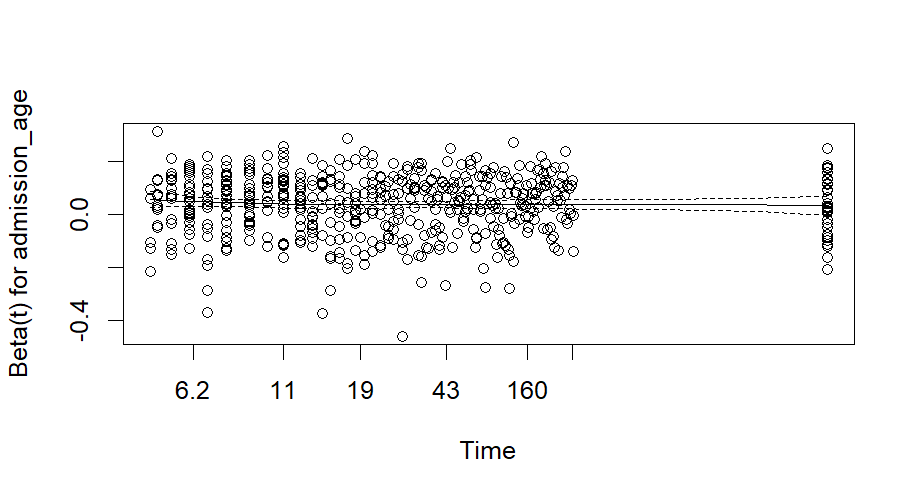

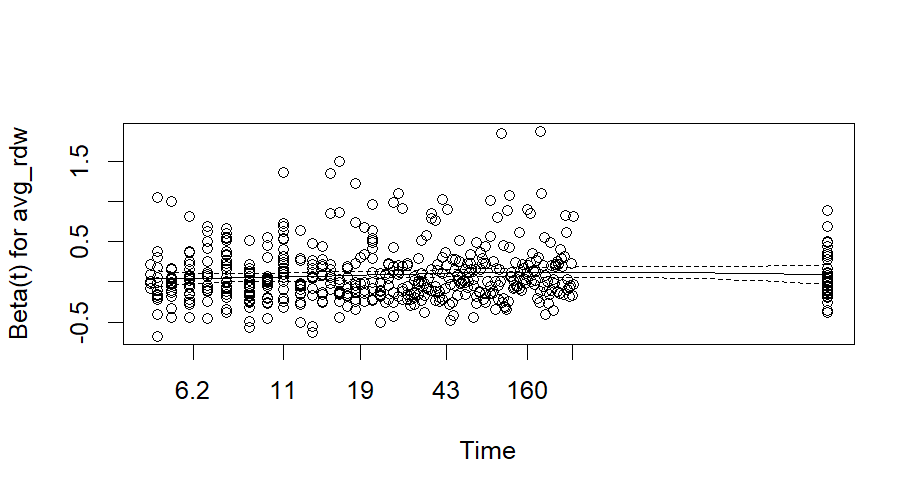

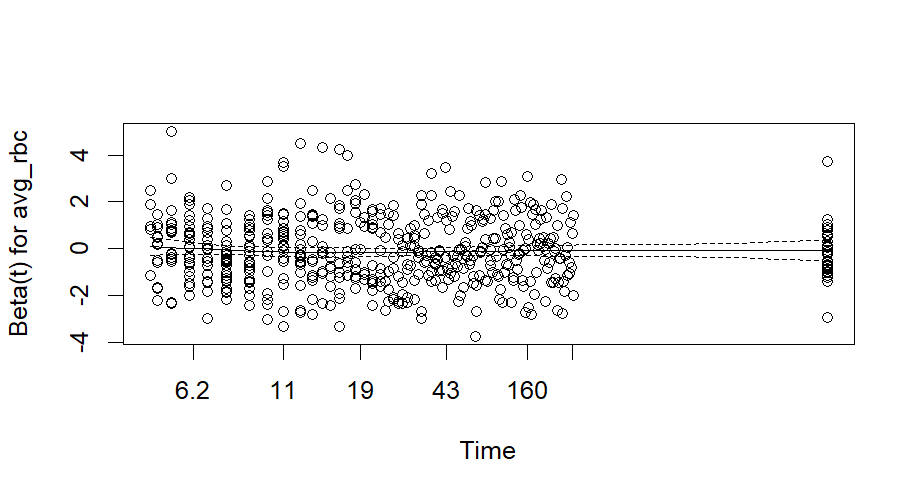

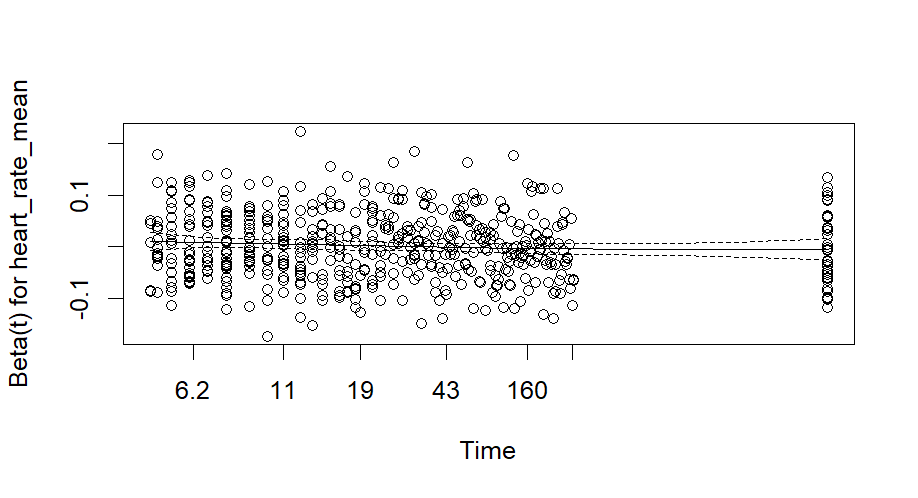

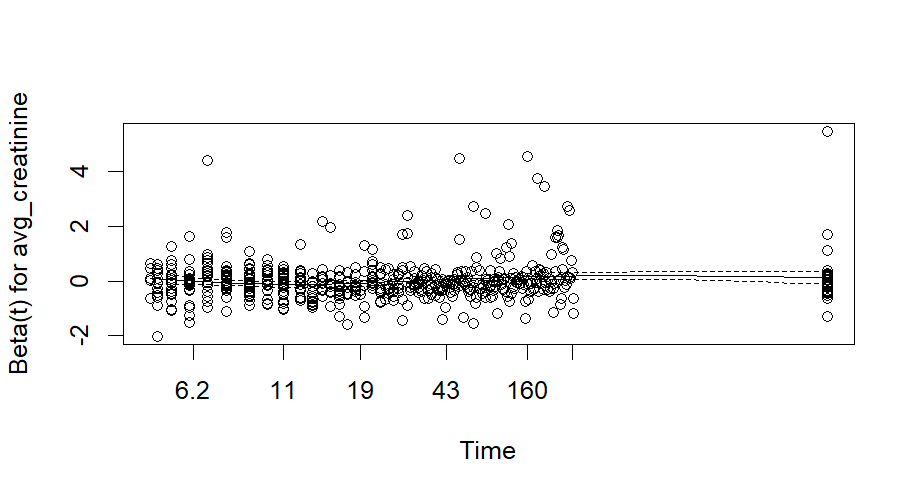

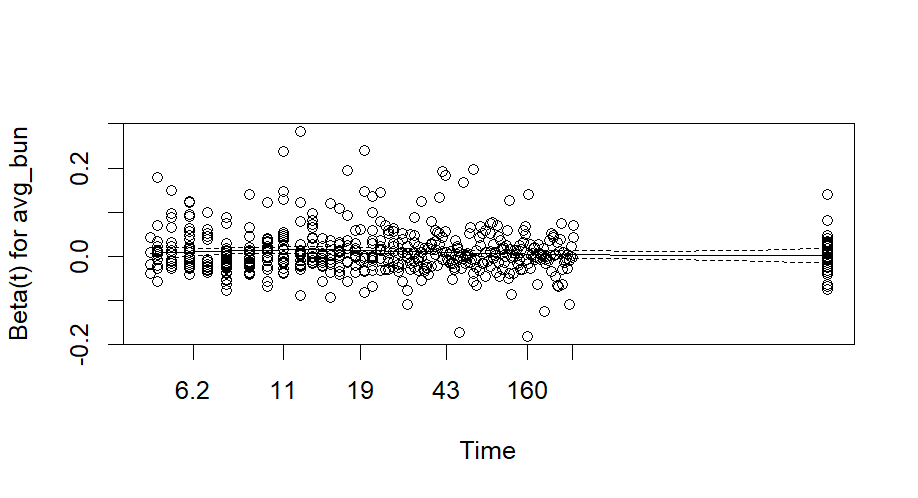

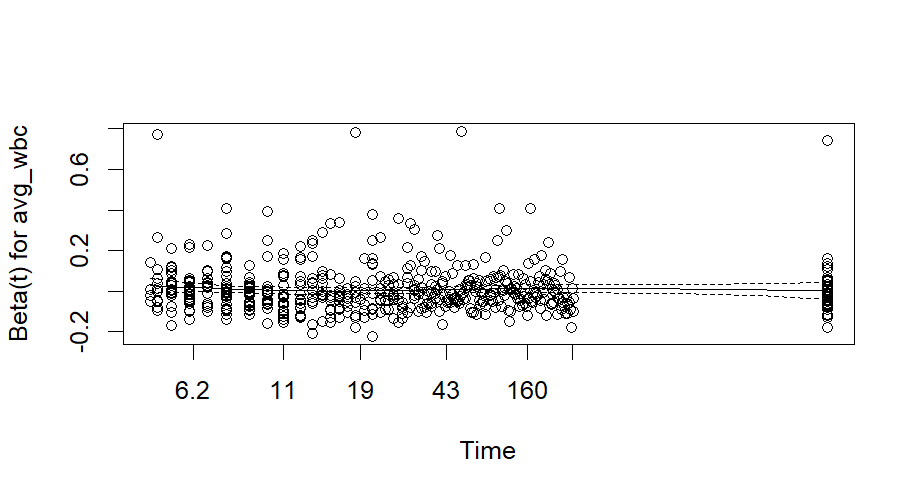

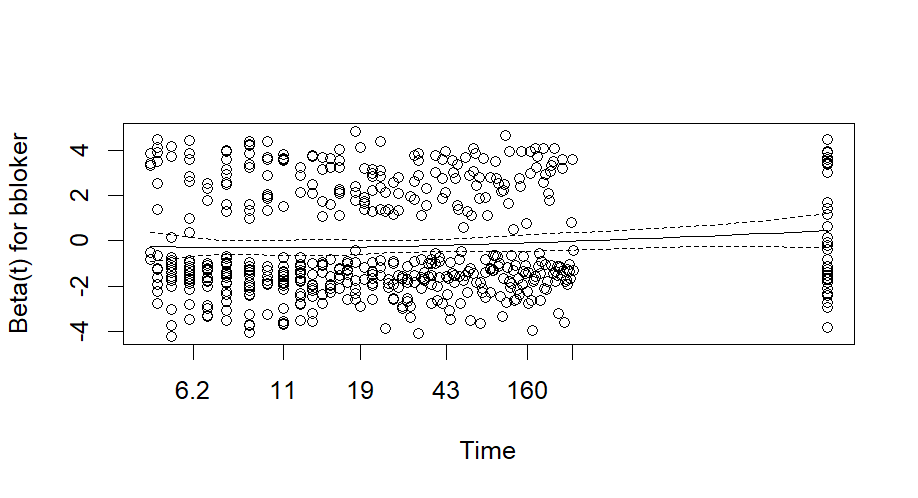

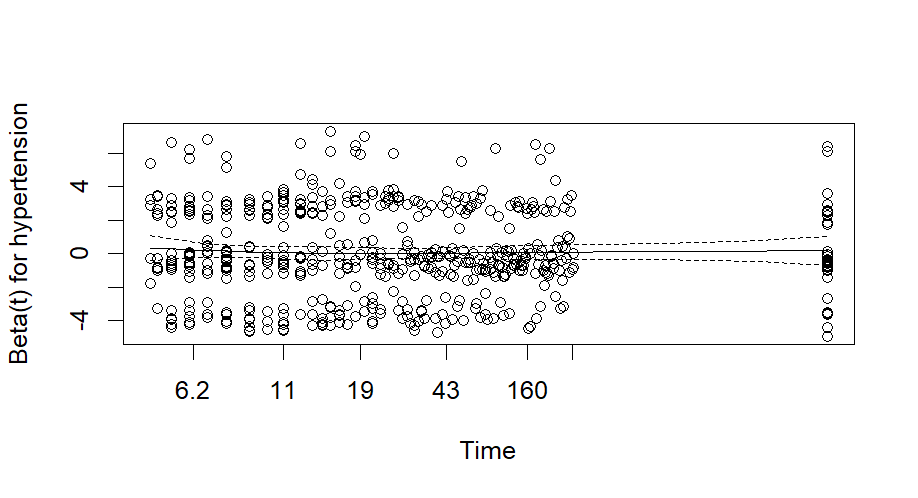

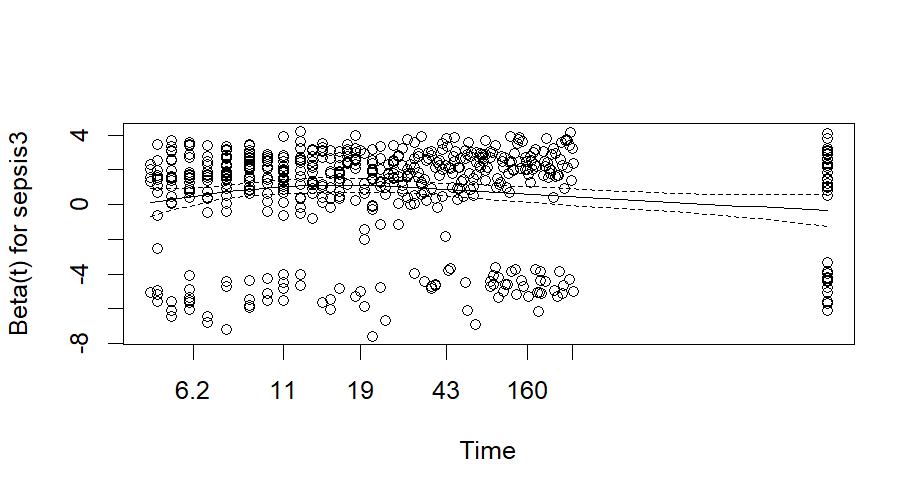

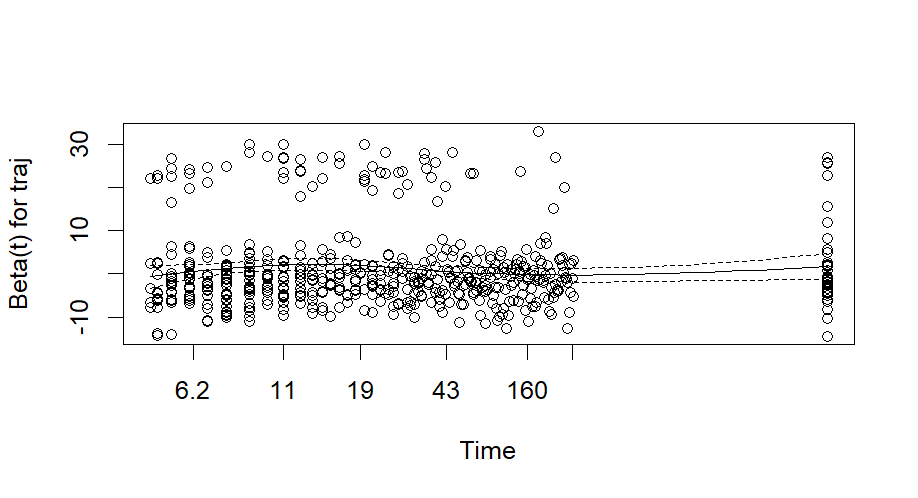


All adjusted covariates : Amiodarone, Congestive heart failure, Liver disease, Diabetes, Statin, Sepsis3, Hypertension, Beta-blocker, WBC, RBC, BUN, RDW, Creatinine, traj

**Supplementary Fig.2** Subgroup analysis of the associations between TyG index trajectories and 90-day all-cause mortality.


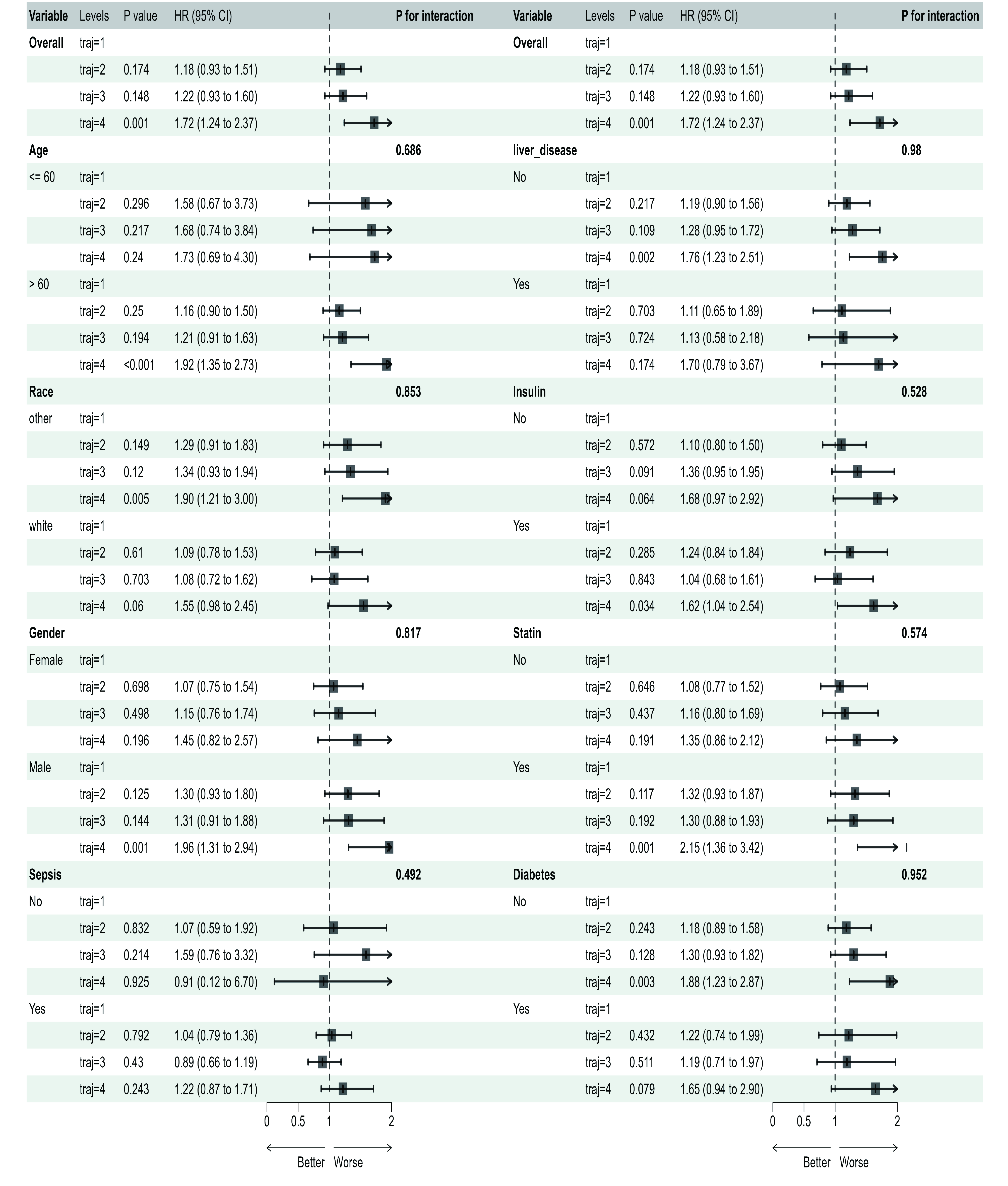


Note: traj1, stable-low group; traj2, slowly ascend group; traj3, ascend-descend group; traj4, fluctuate-high group;

**Supplementary Fig.3** Subgroup analysis of the associations between TyG index trajectories and 180-day all-cause mortality.


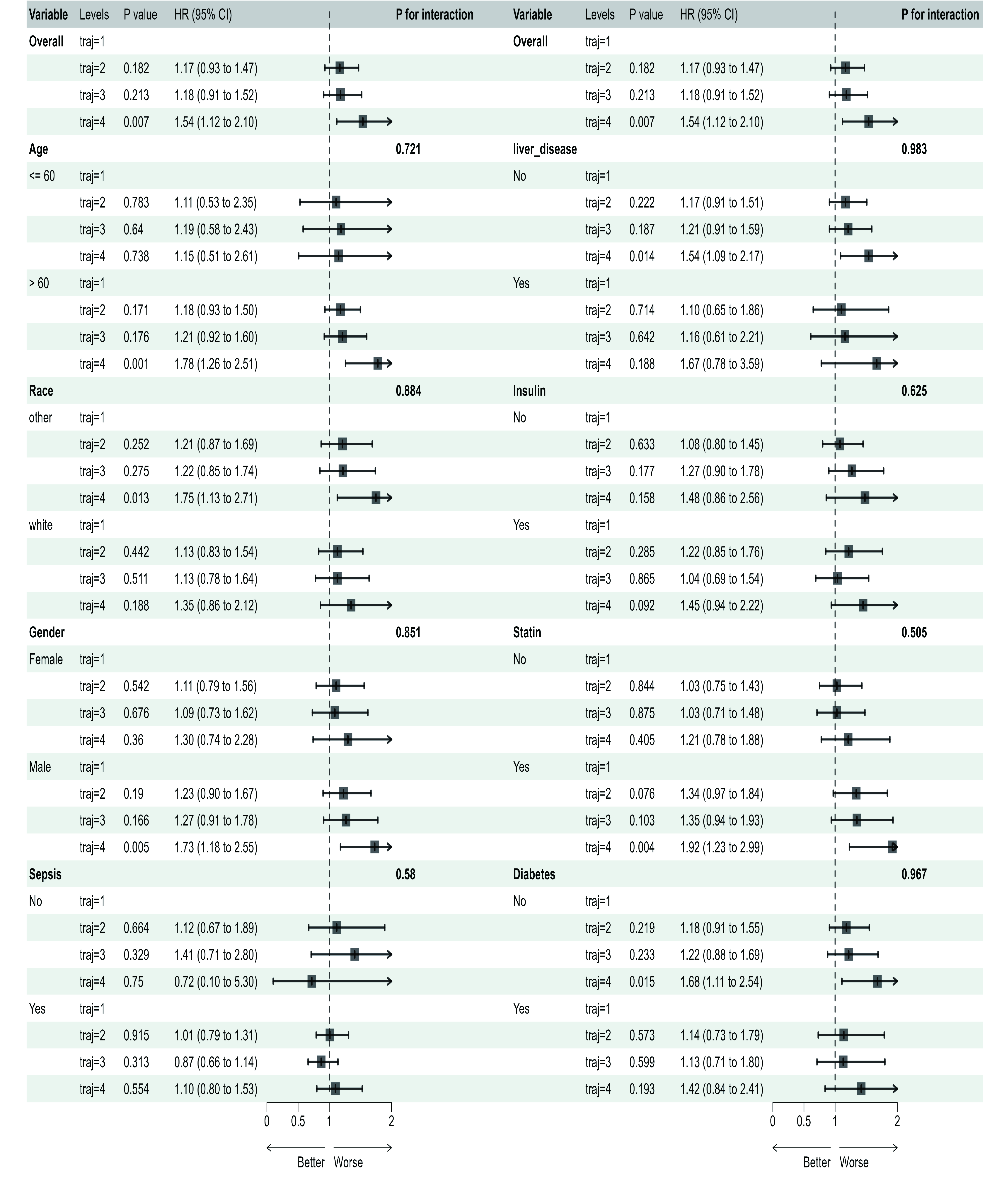


Note: traj1, stable-low group; traj2, slowly ascend group; traj3, ascend-descend group; traj4, fluctuate-high group;

**Supplementary Fig.4** Kaplan–Meier survival analysis for all-cause mortality at 90-day among each triglyceride-glucose (TyG) index trajectory.


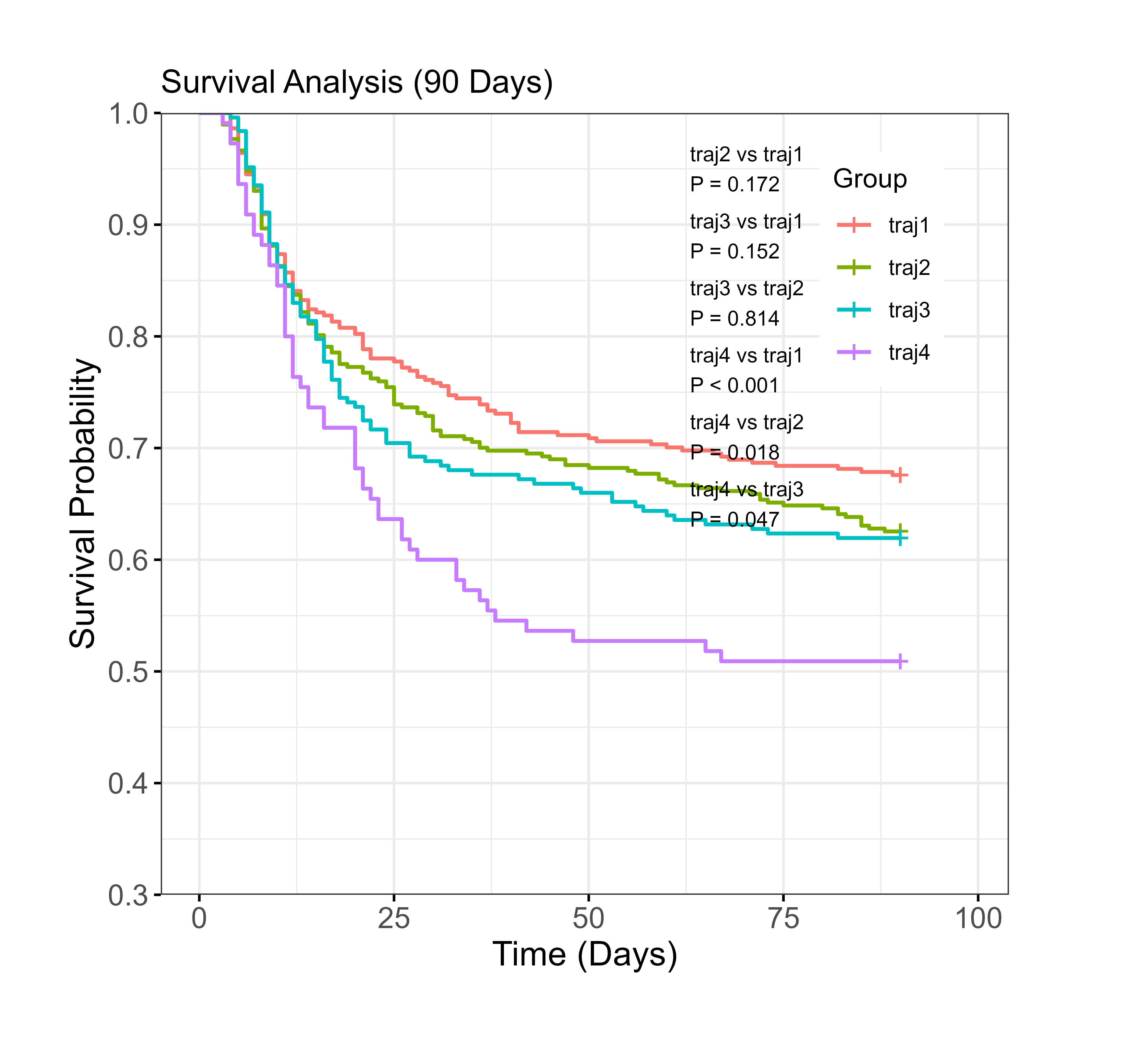


Note: traj1, stable-low group; traj2, slowly ascend group; traj3, ascend-descend group; traj4, fluctuate-high group;

**Supplementary Fig.5** Kaplan–Meier survival analysis for all-cause mortality at 180-day among each triglyceride-glucose (TyG) index trajectory.


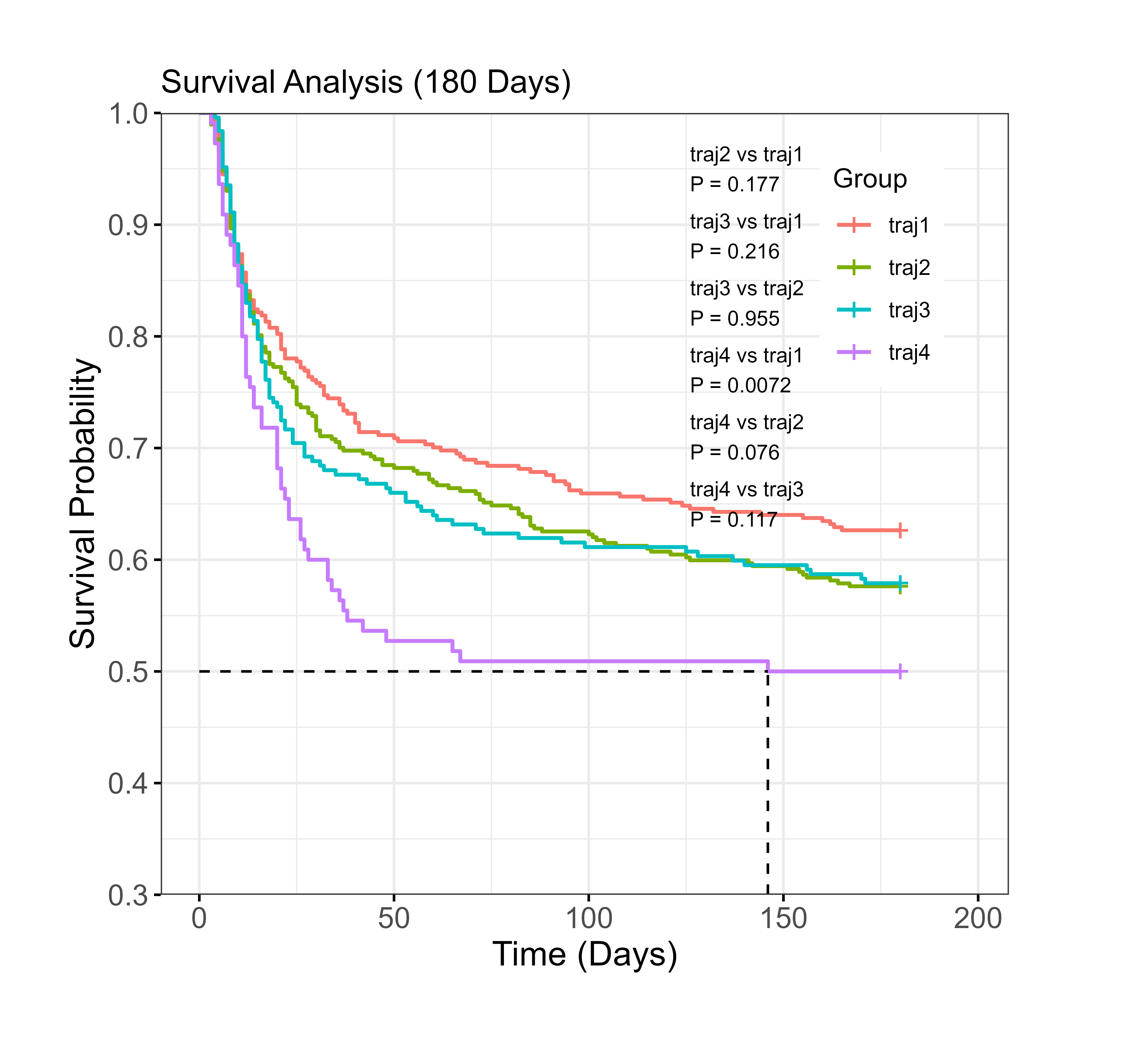


Note: traj1, stable-low group; traj2, slowly ascend group; traj3, ascend-descend group; traj4, fluctuate-high group;

**Relevant code:**

**Sepsis:**

CREATE TABLE spesis3_min_af AS(

SELECT i.subject_id,i.icu_intime,i.stay_id,c.sepsis3

FROM base_min_icu_af i

INNER JOIN mimiciv_derived.sepsis3 c ON i.subject_id=c.subject_id AND i.stay_id=c.stay_id

WHERE c.sepsis3='t'

)

**Warfarin:**

CREATE TABLE warfarin_min_af AS(

SELECT i.subject_id,i.hadm_id,i.icu_intime,i.stay_id,

CASE WHEN sum(c.amount) is not NULL THEN 1

ELSE 0

END AS war

FROM base_min_icu_af i

LEFT JOIN mimiciv_icu.inputevents c on i.subject_id=c.subject_id AND i.stay_id=c.stay_id

WHERE c.itemid in('225913')

AND c.starttime BETWEEN i.icu_intime AND i.icu_outtime

GROUP BY i.subject_id,i.hadm_id,i.stay_id,i.icu_intime

)

**Insulin:**

CREATE TABLE insulin_min_af AS(

SELECT i.subject_id,i.hadm_id,i.icu_intime,i.stay_id,

CASE WHEN sum(c.amount) is not NULL THEN 1

ELSE 0

END AS insulin

FROM base_min_icu_af i

LEFT JOIN mimiciv_icu.inputevents c on i.subject_id=c.subject_id AND i.stay_id=c.stay_id

WHERE c.itemid in('223257','223258','223259','223260','223261','223262','226222','229299','229619')

AND c.starttime BETWEEN i.icu_intime AND i.icu_outtime

GROUP BY i.subject_id,i.hadm_id,i.stay_id,i.icu_intime

)

**Hypertension:**

CREATE TABLE hypertension_af AS

WITH t1 AS (

SELECT d.subject_id, d.hadm_id

FROM mimiciv_hosp.diagnoses_icd d

WHERE d.icd_code IN ('I15', 'I10', '4011', '4019')

)

SELECT

t1.subject_id,

t1.hadm_id,

i.stay_id,

CASE

WHEN t1.subject_id IS NOT NULL THEN 1

ELSE 0

END AS hypertension_with_icu_flag

FROM t1

inner JOIN base_min_icu_af i

ON t1.subject_id = i.subject_id AND t1.hadm_id = i.hadm_id;
